# The impact of elevated temperature and CO_2_ on growth, physiological and immune responses of *Polypedates cruciger* (common hourglass tree frog)

**DOI:** 10.1186/s12983-019-0348-3

**Published:** 2020-01-13

**Authors:** W. A. Manasee T. Weerathunga, Gayani Rajapaksa

**Affiliations:** Department of Zoology and Environmental Management, Faculty of Science, University of Kelaniya, Dalugama, Sri Lanka

**Keywords:** Climate change, Anuran amphibians, Morphometrics, Motility, Physiological responses, Immune response, Antioxidants, Deformities

## Abstract

**Background:**

Amphibians are one of the most susceptible groups to climate change as their development occurs in aquatic environments or in microhabitats with high humidity. Accordingly, our primary objective was to investigate the chronic physiological responses seen in early larval to adult stages of *Polypedates cruciger* (Common hourglass tree frog) to future climate change based on continuous exposure to elevated temperature and elevated CO_2_ -induced low water pH. Free-swimming and free-feeding tadpoles were observed until metamorphosis under four experimental treatments; two elevated temperatures, one elevated CO_2_ (reduced pH) and a control maintained at ambient temperature (29 °C ± 1 °C) and CO_2_ (pH = 7). Elevated temperature treatments were maintained at 32 °C ± 0.5 °C and 34 °C ± 0.5 °C to represent respectively, the future climate scenarios RCP2.6 (Representative Concentration Pathway 2.6, the ‘base-case’ scenario) and RCP8.5 (‘business-as-usual’ scenario) according to the 5^th^ Assessment Report of the IPCC. Elevated CO_2_ treatment was maintained within the pH range of 5.5–5.6 representing the range expected between RCP8.5 and RCP2.6.

**Results:**

Compared to the control, elevated CO_2_ accelerated phenological progression of tadpoles through Gosner stages, thus resulting in lower body size at metamorphosis. Both elevated temperatures significantly delayed the development and reduced the growth of tadpoles. 100% mortality was observed in 34 °C treatment before metamorphosis (before Gosner stage 36) while all the tadpoles died after metamorphosis (at Gosner stage 46) in 32 °C treatment. Elevated CO_2_ increased tadpole activity, in terms of their swimming speed, while both of the elevated temperatures reduced it compared to the control. Catalase activity increased at elevated CO_2_. Ammonia excretion by tadpoles was decreased by elevated CO_2_, but increased under temperature elevation. Both Elevated CO_2_ and temperature treatments reduced the white blood cell count and its percentage of thrombocytes. Percentages of lymphocytes, monocytes and neutrophils were increased at 32 °C, while lymphocyte percentage and lysozyme activity were increased at elevated CO_2_. Several deformities were observed in tadpoles at elevated temperature and CO_2_.

**Conclusions:**

Elevated temperatures and reduced pH due to elevated CO_2_, being major features of climate change, increase the vulnerability of amphibians, who are already one of the most threatened vertebrate groups. Based on our observations on the model amphibian species *P. cruciger*, increased vulnerability to climate change occurs by reducing their growth, body size and motility while also reducing their immunity and inducing physical deformities. These impacts are highly-likely to reduce the foraging, competitive and reproductive capabilities in their natural habitats. We conclude further that even the ‘best-case’ scenario of future climate change can impose significant physiological impacts that could threaten amphibian populations on broader spatial and temporal scales.

**Graphical abstract:**

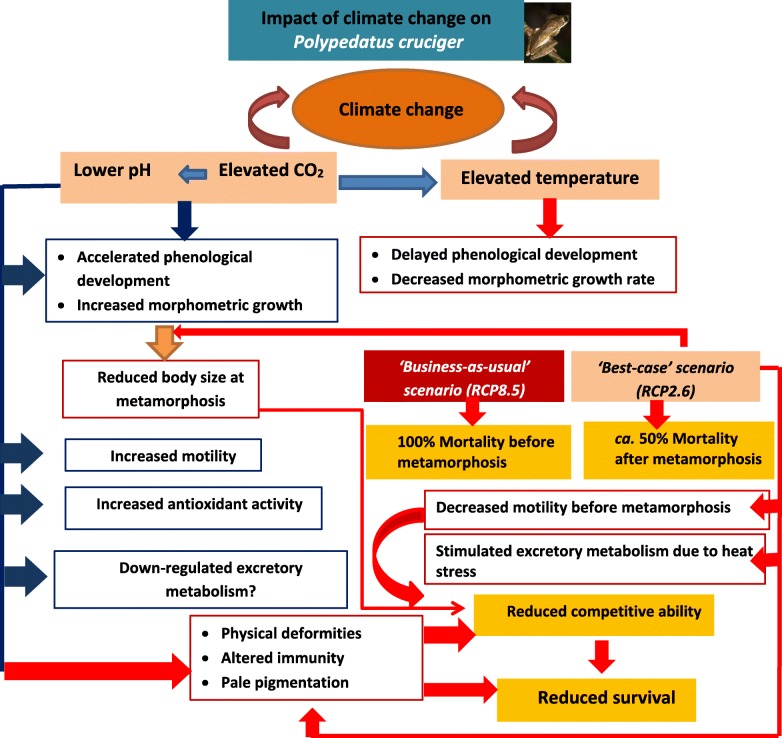

## Background

Anthropogenic emission of carbon dioxide (CO_2_) is widely recognized as the principal cause of rising atmospheric temperatures and long-term climate change [1]. According to the Intergovernmental Panel on Climate Change (IPCC)'s Representative Concentration Pathway 8.5 (RCP8.5), which represents the most fossil fuel-intensive developmental pathway, global atmospheric CO_2_ (C_a_) concentration is projected to increase up to 970 ppm by 2100 [2]. Even under the most environmentally-friendly scenario of RCP2.6, C_a_ is predicted to increase up to 490 ppm by 2050 before stabilization and subsequent decrease towards the end of this century. Absorption of CO_2_ by water bodies such as oceans and lakes increases with increasing C_a_ leading to increased concentrations of dissolved CO_2_ in water and reduced pH. By 2100, pH of ocean surface water is projected to decrease by 0.065 and 0.31 under RCP2.6 and RCP8.5 respectively [1]. Reductions of similar magnitude have been predicted for the pH of freshwater bodies (e.g. Laurentian Great Lakes) as well when increasing atmospheric partial pressure of CO_2_ is considered as the only climate forcing [3]. However, pH of freshwater aquatic environments could decrease further due to CO_2_ generated from decomposing organic matter [4]. Increasing concentrations of CO_2_ and other greenhouse gases enhance the natural greenhouse effect and accelerate global warming [5]. Consequently for all four RCP scenarios, global mean surface temperature (GMST) is projected to increase by 0.3–0.7 °C during 2016–2035 relative to 1986–2005 [1]. Longer-term projections of warming diverge among RCPs, ranging from 0.3–1.7 °C for RCP2.6 to 2.6–4.8 °C for RCP8.5 by 2081–2100 relative to 1986–2005 [1].

Every biological process shows its optimal performance within a specific range of environmental conditions [6, 7]. Thus, shifts in environmental conditions due to climate change may exert a significant influence on biological systems, at the individual as well as ecosystem level. Although the predicted increase in temperature and decrease in pH due to climate change appear small in magnitude, they could have appreciable impacts on biological systems from species to ecosystem levels [8–10]. To assess the impacts of climate change on biological systems, knowledge on three major aspects is required. They are the current climatic conditions and future climatic scenarios, how close organisms are to their tolerance limits in nature; and the degree to which organisms are able to adjust or acclimatize their sensitivity to variations in key climatic parameters [11, 12]. Most research on the response of biological systems to climate change has been based on a few well-studied model organisms such as *Drosophila* sp. and fish species, particularly temperate fish such as salmons and summer flounder *Paralichtys dentatus* [13–15]. Nevertheless, several studies have focused on amphibians, arguably the most endangered group as one third of all extant amphibian species are threatened with extinction [16].

Inhabitation of temporary aquatic habitats is a major factor that makes amphibians highly susceptible to climate change [17]. Being the link of transition of life from water to land, it is a common characteristic of most amphibians to spend at least one stage of their life cycles in water. Often, it is one of the early life stages that is spent in aquatic habitats. Most amphibians live in close proximity to aquatic habitats and return to water for reproduction. Dramatic fluctuations of temperature and pH occur in the temporary and shallow microhabitats that most amphibians and other ectotherms dwell, causing changes in development rates and development cycles [18, 19]. These fluctuations have detrimental impacts on various biological processes at different levels of biological organization, ranging from specific biochemical reactions within an individual organism to ecological interactions of species and communities [7, 20, 21]. As such loss of conducive habitats due to climate change could be the most probable reason for the rapid decline of amphibian population during recent times [22].

Generally, the rate of physiological processes relate non-linearly to temperature and pH [23, 24]. Although organisms are capable of surviving chronically lethal temperatures and pH for short periods, their growth and development depend on a range of cellular processes that require a specific set of environmental conditions (e.g. temperature, pH etc.) over a longer period for optimum performance [25]. Disruption of key cellular processes due to the absence of favourable environmental conditions appear externally as reduced growth rates and activity, delayed or disrupted development, leading to increased vulnerability to predation, risk of infection and desiccation. Consequently, climate change is regarded as a principal cause of the decline of amphibian and other ectothermic populations worldwide [26, 27].

In living organisms, respiration is a continuous process and reactive oxygen species (ROS) are generated simultaneously. Commonly generated ROS include superoxide and hydroxyl radicals and hydrogen peroxide (H_2_O_2_) [28, 29]. Catalase is a universal enzyme present in all aerobic organisms. It is capable of breaking down H_2_O_2_ to water and oxygen [30]. All enzymes have an optimal activity range of temperature and pH, Hence, changes in temperature and pH in the external microhabitat could affect enzymatic antioxidant defence systems. Optimum temperature and pH ranges for catalase enzyme activity are species-specific [31, 32]. Accordingly, the activity of antioxidant defense enzymes such as catalase requires investigation in amphibians when they are exposed to increasing temperature and decreasing pH in their microhabitat.

Emergence of new amphibian diseases and increasing severity of prevalent diseases has been reported worldwide and environmental factors are suspected to be their cause [33, 34]. This also has probably contributed to the worldwide decline of amphibian populations [35–38]. Recent extinction of many tropical frog species could be due to increased infection risk associated with climate change [39]. Accordingly, we investigated the immune response to varying climate change parameters by quantifying the response of white blood cells (WBCs) and lysozyme activity to elevated temperature and CO_2_. White blood cells play a major role in determining the immunity of living systems [40]. Lysozyme is an enzyme involved in bacterial lysis. Therefore, lysozyme activity is an indicator of immune status of an organism [41]. Lysozyme is known to be stable in temperatures as high as 72 °C and has an optimum activity within the pH range from 6 to 9 [42, 43].

Most research on the impact of environmental variations on immunity of amphibians have involved lower temperatures such as the immune response of amphibians to winter and hibernation [44]. In addition, some research has focused on immune response of ectotherms such as fish to stress caused by handling, transportation, and overcrowding [45]. Apart from the work of Bibi and Christi [46], research on the impacts of elevated temperature and fluctuating acidity on immunity, especially of tropical amphibian species, is limited. Hence, the current study will be important in setting a baseline for tropical species.

Developmental and morphological abnormalities associated with viscera, mouth, eye, and limbs of amphibians are common when exposed to environmental stressors during their developmental stages [47]. Although most research has focused on deformities caused by exposure to toxicants such as pesticides and heavy metals [48, 49], impacts of increasing temperature and acidity on development of deformities is an area that has not been studied extensively.

Sri Lanka is a country rich in amphibian diversity, particularly in anuran diversity [50]. So far, 103 species have been described out of which 87 are endemic to the island [50]. Being a tropical country, it is possible that impacts of climate change on anuran diversity of Sri Lanka would be lower compared to that of temperate regions [51, 52] as the higher latitudes have experienced greater warming than the lower latitudes [53, 54]. On the other hand, tropical species do not usually encounter seasonal changes. Hence they are mostly adapted to constant environmental conditions that are usually close to their physiological optima [55–57]. Such species may have limited acclimatizing capacity as they do not encounter seasonal changes [12]. Therefore, tropical ectothermic species such as anurans have limited behavioural and physiological adaptations to adjust to changing climatic factors [58]. This makes tropical species more vulnerable to even small changes of climatic factors [59]. Excessive warming during recent years, with 2016 and 2017 being reported as the warmest years on record, and the tropical region being the warmest part of the world, tropical ectotherms are highly likely to be at risk [59]. Even though Sri Lanka is an amphibian hotspot, research on the impact of climate change on physiology of amphibians is scarce. These facts emphasize the need for research on the potential physiological impacts of climate change on a tropical endemic amphibian species.

Accordingly, our overall objective was to determine the chronic physiological responses of *Polypedates cruciger* to climate change simulated as long-term, continuous exposure to elevated temperature and elevated CO_2_ (and consequently reduced pH) from early larval to adult stages. Specifically, we focused on the responses of following processes and parameters: (a) Survival and time taken for metamorphosis; (b) Growth and morphometrics of tadpoles; (c) Activity of tadpoles measured in terms of swimming performance; (d) Immunity as measured by differential white blood cell counts and lysozyme activity; (e) Antioxidant capability as indicated by catalase activity; (f) Excretory metabolism measured as ammonia released.

## Material and methods

### Organism used in the study

The model organism used for this study was *Polypedates cruciger,* (Anura: Ranidae), the Common Hourglass Tree Frog. It is an arboreal species that is usually found in forests of both wet and dry zones of Sri Lanka up to an altitude of 1525 m above mean sea level. The geographical selectivity of this species to higher altitudes makes it an excellent model for this study, as such species are considered to be more sensitive to environmental fluctuations, especially in temperature [59]. It usually spawns in a foam nest attached to a surface (e.g. tree branch, pond bank) few inches above a shallow water surface. Hatched tadpoles fall to the water below and carry out their development therein. Recent studies show that *P. cruciger* has expanded its habitat range, being dominant in human-modified environments [60]. Reproductive behavior of this species also makes it ideal for the current study because the shallow temporary water bodies in which it usually reproduces and tadpoles develop are highly susceptible to temperature and pH fluctuations. The current IUCN conservation status of *P. cruciger* is Least Concerned (LC) which makes it convenient to use as a model organism for scientific research.

### Sample collection

We collected foamy egg clutches from home gardens in Kandy, Sri Lanka (Latitude 7.2906^o^N; Longitude 80.6337°E). They were transported to the Department of Zoology and Environmental Management, University of Kelaniya, Sri Lanka. The egg clutches were placed in 15 Litre glass aquaria containing dechlorinated tap water. The tanks were placed in a naturally-ventilated room within a building. The diurnal variation of air temperature followed a pattern similar to that of ambient temperature in the external environment. In addition to natural sunlight, artificial light was provided during the day time by fluorescent bulbs. Standard keys were used in species identification of tadpoles [61]. Once they reached the free-feeding and free-swimming stage, we pooled all tadpoles from all egg clutches and assigned fifteen tadpoles randomly to each treatment tank using a pasteur pipette. Each tank contained dechlorinated tap water (5 L) and tadpoles were acclimated to tanks for 1 week. Tadpoles were fed twice daily alternatively with formulated fish feed and chopped spinach leaves ad libitum [62].

### Experimental setup

Our experimental setup included four treatments consisting of two elevated temperature treatments, one elevated CO_2_ treatment and a control treatment at ambient temperature and CO_2_. In the elevated CO_2_ (ECO2) treatment, we bubbled CO_2_ into each assigned tank until pH of water was in the range of 5.5–5.6. The pH of freshwater should be 5.5 and 5.6 respectively corresponding to the atmospheric CO_2_ concentrations (C_a_) projected under RCP8.5 and RCP2.6 pathways. However, one pH (i.e. higher dissolved CO_2_) treatment in the range of 5.5–5.6 was maintained as precise maintenance of two separate pH levels in close range was not possible. Sodium citrate-citric acid biological buffer was used to maintain pH within the required range [45] and to control the solubility of CO_2_ in water.

We maintained our control (i.e. ambient temperature and CO_2_) treatment at an ambient temperature of 29 °C ± 1 °C and a pH of 7.0. In the two elevated temperature treatments, we increased water temperature by fitting Atman-100 W (Atman, China) aquarium heaters. We maintained water temperature at 32 °C (ETem32) and 34 °C (ETem34), which represented predicted temperature increases of 2 °C and 4 °C by 2100 under the IPCC’s ‘best-case’ scenario (RCP2.6) and the ‘business-as-usual’ scenario (RCP8.5) respectively. In both elevated temperature treatments, we increased water temperatures gradually at the rate of 1 °C per day up to the respective temperatures. The daily temperature fluctuation was 0.5 °C in both treatments. We triplicated each treatment. We fixed aerators bubbling ambient air containing O_2_ to all tanks, except those containing the elevated CO_2_ treatment. Despite not receiving bubbled ambient air, we expected that the elevated CO_2_ treatment received adequate oxygen via dissolution from ambient air. We replaced water every 48 h by siphoning and refilling with an equal volume of de-chlorinated water while maintaining all tanks at natural photoperiod.

### Measurements

#### Morphometrics of tadpoles

We measured morphometrics of a sample of three tadpoles selected randomly from each tank once a week by placing a graph sheet underneath the tank. Length was measured by taking a freeze frame photograph and measuring it using ImageJ software (Version 1.51 k, National Institute of Health, USA). Total body length, snout-vent length, tail length, and body width were measured in tadpoles.

#### Activity of tadpoles

We used swimming speed to assess the activity of tadpoles using the method adopted by Jung and Jagoe [63]. We selected three tadpoles randomly from each tank for this measurement, which was performed fortnightly. We used a narrow channel (60 cm) filled with water taken from the tank in which a selected tadpole was present. Next we placed the tadpole at one end of the channel and prodded its tail gently with a pipette tip. Then we measured the time taken for the tadpole to swim the channel distance using a stopwatch.

#### Ammonia excretion

We measured the excretory metabolism of tadpoles in terms of their ammonia excretion to the water by the Phenate method at weekly intervals [64]. Briefly, to a water sample (25 ml), phenol solution (1 ml), sodium nitroprusside solution (1 ml) and oxidizing solution (2.5 ml) was added and after standing for 1 h for colour development, absorption was measured at 640 nm using UV and visible absorption spectrophotometer (Cecil, Great Britain). Ammonia concentration of each sample was determined using ammonium chloride as the standard.

#### Metamorphosis and mortality

We observed the tanks daily for dead tadpoles. Time taken for 50% of tadpoles to reach the stages of hind limb emergence (Gosner Stage 36–39) and fore limb emergence (Gosner Stage 42–46) were determined by daily observation. The experiment was terminated once 50% of tadpoles in a tank reached Gosner Stage 46.

#### Hematological analysis

Once 50% of tadpoles reached Gosner Stage 46, three tadpoles were randomly selected from each tank. They were euthanized using benzocaine (Sigma-Aldrich) (1.21 mM) and blood was drawn by heart puncture using a micropipette [65]. A blood smear was prepared and was stained using the Leishman-Wright’s stain [66]. Then we performed a differential white blood cell count (WBC) for each slide by determining the percentage of neutrophils, eosinophils, basophils, monocytes, lymphocytes, and thrombocytes in 50 WBC counted [65, 67–69]. Also we counted the ratio of WBC per 2000 erythrocytes in each slide as in Schermer [68].

#### Catalase enzyme activity

We measured the catalase activity in terms of the rate of H_2_O_2_ degradation by catalase [70]. We stored the liver extracted from each euthanized adult in 20 mM Tri-HCl buffer (Tris-HCl 20 mM, EDTA 1 mM, DL-dithiothreitol (DTT) 1 mM, sucrose 0.5 M, KCL 0.15 M, phenylmethylsulfonyl fluoride (PMSF) 1 mM), pH 7.4, in -80 °C. The preserved liver tissues were weighed and homogenized in 50 mM phosphate buffer (PB) (pH 7.4). Homogenate was centrifuged at 5000G in 4 °C for 15 min and the supernatant was separated. Lysate samples were diluted to 1:50 with PB and mixed with H_2_O_2_ (10 mM, 1 mL) initial absorbance at the wavelength 240 nm was measured immediately by spectrophotometer (Nanospec, Shimadzu, Japan). Decrease of absorbance was measured after 4 min. We used the formula of Cuellar-Cruz et al. to calculate the catalase activity [71]. Protein concentration of the sample was measured by Bradford assay [72]. Catalase activity was quantified relative to the protein concentration and expressed in terms of units per mg of protein.

#### Stomach enzyme activity

Stomachs from dissected tadpoles were preserved in phosphate-buffered saline (PBS) (pH 7.4) in -20 °C. Weight of each stomach sample was measured and was homogenized in PBS (100 μL). Using the homogenate, activity of stomach lysozyme activity was assessed using the lysoplate method [73]. Briefly, 20 μl of the homogenate was placed into wells (3.5 mm diameter and 4 mm deep) cut on nutrient agar in petri dishes with 100 mm diameter. The nutrient agar was amended with a *Micrococcus luteus* culture (500 μl of the bacterial culture having a cell concentration of 1 × 10^6^ cfu/ml was added to 100 ml of nutrient agar). The diameter of the lytic zones was measured 48 h after incubation at 34 °C in nine replicates.

### Statistical analyses

We used two graphical methods, namely, the normal probability plot (P-P plot) and the quantile-quantile plot (Q-Q plot) to test normality of continuous response variables. As the data points did not deviate appreciably from the fitted straight lines in the respective P-P and Q-Q plots, we proceeded with statistical analyses of all continuous variables using analysis of variance.

We determined the significance of treatment effects on the times taken to reach Gosner stages 36–39 and 42–46 by analysis of variance with the effects of elevated CO_2_ and temperature considered as fixed effects. Means were compared using the Duncan’s multiple range test.

We determined the time courses of the variation of morphometrics (i.e. total body length, snout-vent length, tail length and body width) by fitting growth curves using second-order polynomial functions. Growth rates of morphometric characters at Gosner stages 36–39 and 42–46 were computed as the first-derivative of the fitted polynomial functions at the time points of reaching the above stages. Initial growth rates were estimated by extrapolating time to zero. We tested the significance of treatment effects on morphometrics by repeated measures analysis of variance (RM ANOVA) using PROC MIXED of the Statistical Analysis System (SAS) [74]. A fixed effects model having a variance-covariance structure with compound symmetry was used after testing several alternative variance-covariance structures (i.e. unstructured, autoregressive and autoregressive with heterogeneous variances). We tested the significance of individual effects of elevated CO_2_ and elevated temperatures relative to the control (i.e. ambient CO_2_ and temperature) using separate contrasts within the PROC MIXED procedure. Effects of different treatments were compared among themselves using the same procedure. We compared the morphometrics at the two specific Gosner stages 36–39 and 42–46 by selecting the morphometric data at equivalent stages in different treatments and subjecting them to analysis of variance. Least squares means (LSMEANS) were used to test significance of treatment effects on morphometrics at equivalent stages. We tested the effects of elevated CO_2_ and temperature treatments on the activity of tadpoles by applying RM ANOVA to swimming speed data following the same procedure that was used for analysis of morphometric data.

We used categorical data analysis with PROC CATMOD in SAS to determine the significance of treatment effects on the counts of white blood cells (expressed as a percentage out of 2000 red blood cells) and on their different types (expressed as a percentage out of 50 white blood cells). Maximum likelihood estimates of frequencies in a log-linear model were used in PROC CATMOD. Significance of individual treatment comparisons was tested using a Z-statistic calculated from the respective maximum likelihood frequencies and their standard errors.

## Results

### Phenology

**Fig. 1:**
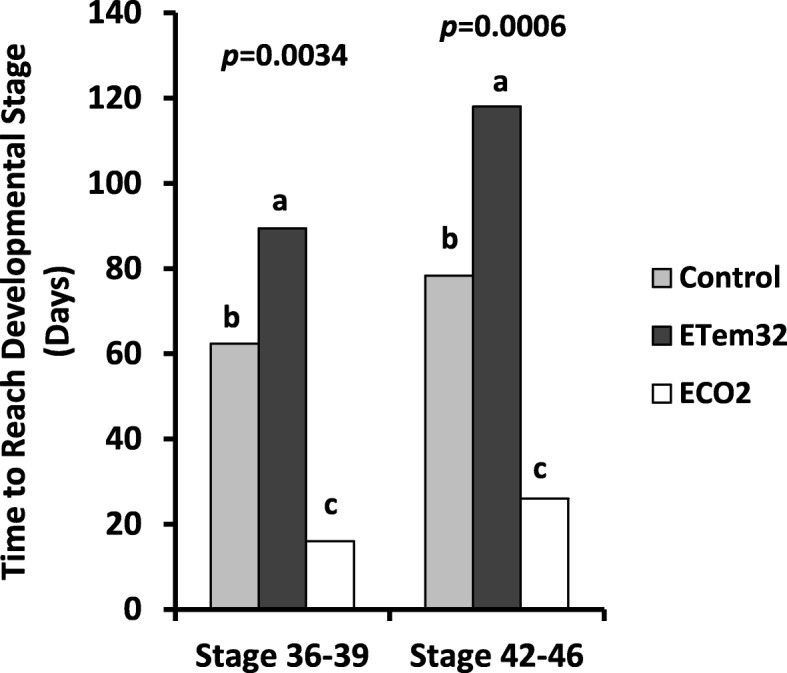
Time to reach specific Gosner stages for 50% of tadpoles experiencing elevated temperature and CO_2_ treatments. Control – Ambient CO_2_ (water pH = 7) and water temperature at 29 ± 1 °C; ETem32 – Water temperature elevated to 32 °C; ETem34 – Water temperature elevated to 34 °C. ECO2 – CO_2_ bubbled to water to maintain pH at 5.5–5.6. Each bar is a least squares mean of three replicate tanks each containing 15 tadpoles. Least squares means with the same letter are not significantly different at *p* = 0.05

Tadpoles in all treatments reached Gosner Stages 26–30 (free feeding and free swimming stage) 2 Weeks After Hatching (2 WAH). In elevated water temperature of 34 °C (ETem34), none of the tadpoles survived until Gosner Stage 36 (hind limb emergence) and all tadpoles died 11 WAH. In the rest of the treatments (i.e. ECO2, ETem32 and the Control), durations to reach Gosner Stages 36–39 and 42–46 were significantly different among treatments (*p* < 0.05) (Fig. 1). Elevation of water temperature to 32 °C (ETem32) delayed development of tadpoles, with tadpoles in ETem32 taking a substantially longer time to reach the two stages than the control.

Tadpoles in the elevated CO_2_ (ECO2) treatment reached both stages earlier than those in the control (*p* < 0.05).

### Growth and morphometrics

**Fig. 2:**
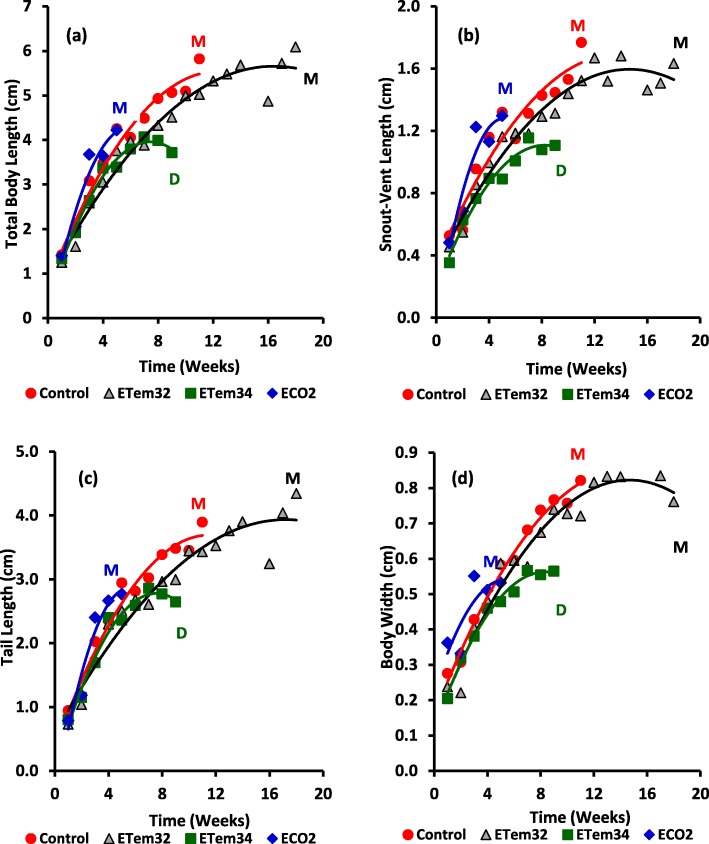
Variation of morphometric characters of tadpoles such as (**a**) Total body length, (**b**) Snout-Vent length, (**c**) Tail length and (**d**) Body width with time after hatching in different treatments. Control – Ambient CO_2_ (water pH = 7) and water temperature at 29 ± 1 °C; ETem32 – Water temperature elevated to 32 °C; ETem34 – Water temperature elevated to 34 °C. ECO2 – CO_2_ bubbled to water to maintain pH at 5.5–5.6. Each data point is a mean of three replicates. M – Metamorphosis; D – Death. Lines indicate second-order polynomial curves. Red circles indicate the Control group. Grey triangles indicate the ETem32 group. Green squares indicate the ETem34 group. Blue diamonds indicate the ECO2 group

Variation of total body length, snout-vent length, tail length, and body width of all treatments showed second order polynomial relationships with time (Fig. 2 and Additional file 1: Table S1). Repeated measures analysis of variance (RM ANOVA) showed highly-significant treatment effects on all morphometric characters (Table 1).

**Table 1:** Significance of treatment effects and time on morphometric characters

^a^Effect	df	Total body length	Snout-vent length	Tail length	Body width
Time	16	< 0.0001	< 0.0001	< 0.0001	< 0.0001
Treatment	3	0.0008	< 0.0001	0.0086	0.0021
Time x Treatment	22	ns	ns	ns	ns

^a^Based on repeated measures analysis of variance (RM ANOVA); ns- Non-significant at *p* = 0.05

While all morphometric characters showed highly-significant variation with time (*p* < 0.0001), the time x treatment interaction effect was not significant (*p* = 0.05). Significance tests for contrasts involving different treatment comparisons showed that there was no significant difference between ECO2 and the control for any of the morphometric characters (Table 2 and Fig. 2). In contrast, morphometrics of tadpoles in elevated temperature treatments, both combined and taken individually, were significantly different from those in the control (*p* < 0.01). Elevated temperatures reduced all morphometric characters relative to the control (Fig. 2). Snout-vent length and body width were lower at ETem34 in comparison to ETem32 (*p* < 0.01). Comparison between the effect of ECO2 and the combined effect of elevated temperatures (both ETem32 and ETem34 included in the contrast) was significant for all morphometric characters except tail length (*p* < 0.05). When effects of the two elevated temperatures were compared separately, comparison between ECO2 and ETem32 was significant only for total body length. On the other hand, the comparison between ECO2 and ETem34 was significant for all characters except tail length. In all instances, where the comparison between ECO2 and elevated temperatures were significant, elevated temperatures had lower morphometrics relative to ECO2 (Fig. 2).

**Table 2:** Significance of individual contrasts comparing different treatment combinations on morphometric characters

Contrast	Total body length	Snout-vent length	Tail length	Body width
Control vs ECO2	ns	ns	ns	ns
Control vs ETem32	0.0075	0.0488	0.0275	ns
Control vs ETem34	0.0022	< 0.0001	0.0089	0.0033
Control vs (ETem32, ETem34)	0.0022	0.0011	0.0087	0.0344
ETem32 vs ETem34	ns	0.0101	ns	0.0220
ECO2 vs ETem32	0.0436	Ns	ns	ns
ECO2 vs ETem34	0.0500	0.0016	ns	0.0321
ECO2 vs (ETem32, ETem34)	0.0227	0.0040	ns	0.0477

Significance of these contrasts was tested in repeated measures analyses of variance carried out using PROC MIXED in SAS for the whole experimental period. Contrasts involving ECO2 involved data during the initial 5 weeks after hatching. Contrasts involving the control and ETem34 involved data during the initial 11 weeks after hatching

**Fig. 3:**
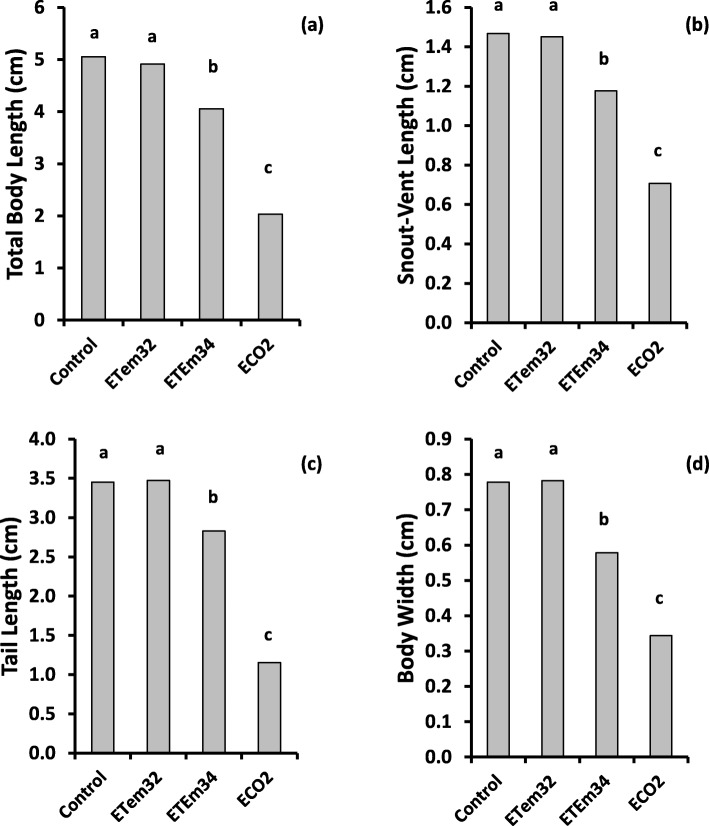
Effects of elevated temperature and CO_2_ treatments on morphometrics of tadpoles such as (**a**) Total body length, (**b**) Snout-vent length, (**c**) Tail length, (**d**) Body width at Gosner stage 36–39. Control – Ambient CO_2_ (water pH = 7) and water temperature at 29 ± 1 °C; ETem32 – Water temperature elevated to 32 °C; ETem34 – Water temperature elevated to 34 °C. ECO2 – CO_2_ bubbled to water to maintain pH at 5.5–5.6. Each bar is a least squares mean of three replicate measurements. Least squares means with the same letter are not significantly different at *p* = 0.05

**Fig. 4:**
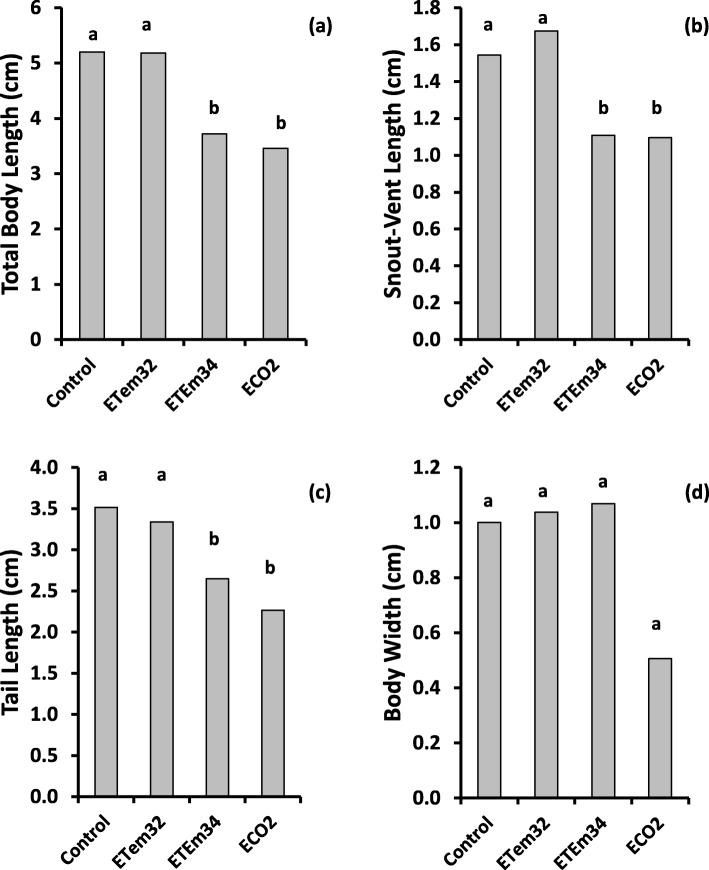
Effects of elevated temperature and CO_2_ treatments on morphometrics of tadpoles such as (**a**) Total body length, (**b**) Snout-vent length, (**c**) Tail length, (**d**) Body width at Gosner stage 42–46. Control – Ambient CO_2_ (water pH = 7) and water temperature at 29 ± 1 °C; ETem32 – Water temperature elevated to 32 °C; ETem34 – Water temperature elevated to 34 °C. ECO2 – CO_2_ bubbled to water to maintain pH at 5.5–5.6. Each bar is a least squares mean of three replicate measurements. Least squares means with the same letter are not significantly different at *p* = 0.05

Comparison of morphometric characters at equivalent developmental stages (i.e. Gosner stages 36–39 and 42–46) showed highly-significant variation among experimental treatments (*p* < 0.001). At both stages, ETem32 did not cause significant changes in morphometric characters in comparison to the control (Figs. 3 and 4). However at Gosner stage 36–39, ECO2 reduced all morphometric characters significantly below those of the control and ETem32. At Gosner stage 42–46 also, ECO2 caused similar reductions of all morphometric characters except body width. Even though tadpoles developing in ETem34 died before reaching Gosner stage 36–39, their morphometrics 7 and 9 WAH were used to make comparisons with morphometrics of other treatments at Gosner stages 36–39 and 42–46 respectively. All morphometric characters of tadpoles in ETem34 at 7 WAH were lower than the corresponding values of tadpoles in the Control and ETem32 at Gosner stage 42–46, but were higher than those in ECO2 (Fig. 3). Total body length and snout-vent length of tadpoles in ETem34 at 9 WAH (at the time of their death) were lower than the corresponding values in the Control and ETem32 at Gosner stage 42–46 (Fig. 4). In contrast, the corresponding values of tail length and body width of tadpoles in ETem34 were not significantly different from those of the control and ETem32. On the other hand, morphometrics of tadpoles in ETem34 at 9 WAH and those in ECO2 at Gosner stage 42–46 were similar (Fig. 4). This was different from the corresponding comparison at the earlier stage (7 WAH and Gosner stage 42–46) (Fig. 3).

At Gosner stage 36–39, both elevated temperatures reduced growth rates of all morphometric characters in comparison to the control (Additional file 2: Table S2). A similar observation was made at Gosner stage 42–46 also, with the exception of snout-vent length and tail length in ETem32. In contrast, morphometric growth rates in ECO2 were greater than in the control at both stages. Similarly, ECO2 increased estimated initial growth rates of all morphometric traits above those of the control. In contrast, no consistent differences could be observed between initial growth rates of the elevated temperature treatments and the control.

### Activity of tadpoles

**Fig. 5:**
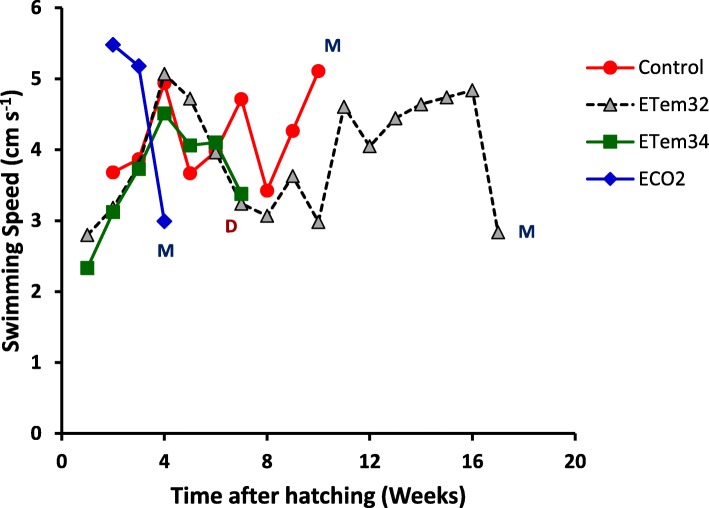
Variation of swimming speed of tadpoles in different treatments with time. Control – Ambient CO_2_ and water temperature at 29 ± 1 °C; ETem32 – Water temperature elevated to 32 °C; ETem34 – Water temperature elevated to 34 °C. ECO2 – CO_2_ bubbled to water to maintain pH at 5.5–5.6. M – Metamorphosis; D - Death

The comparative variation among treatments of tadpole activity (Fig. 5) differed at different times after hatching. This was shown as a highly-significant treatment x time interaction effect in the RM ANOVA (*p* < 0.0001). In addition, there were significant treatment (*p* = 0.0126) and time (*p* = 0.0038) effects on swimming speed. Because of the significant treatment x time interaction, treatments were compared at each time point separately. During 2^nd^ and 3^rd^ WAH, tadpoles in ECO2 had faster swimming speeds than the control and the elevated temperature treatments (Table 3).

**Table 3:** Significance of contrasts comparing the swimming speed of tadpoles at elevated CO_2_ with other treatments

Contrast	Weeks after hatching		
	2	3	4
ECO2 vs Control	0.0005	0.0220	0.0072
ECO2 vs ETem32	0.0003	0.0432	0.0046
ECO2 vs ETem34	0.0004	0.0201	0.0408
ECO2 vs (ETem32, ETem34)	0.0597	0.0147	0.0067

Significance of these contrasts was tested in analyses of variance carried out separately for each week using PROC GLM of SAS

However, their swimming speed slowed down substantially during the 4^th^ week as they neared metamorphosis in the 5^th^ week. Consequently in the 4^th^ week, activity of tadpoles in ECO2 was significantly lower than that in all other treatments. In contrast to the variation pattern shown in ECO2, activity of tadpoles in the rest of the treatments increased up to the 4^th^ week (Fig. 5). At the early stage (i.e. 2^nd^ week), swimming speed of tadpoles in the two elevated temperature treatments were significantly lower than in the control (Table 4).

**Table 4:** Significance of contrasts comparing tadpole activity in elevated temperature treatments with those in other treatments

Contrast	Weeks after hatching							
	2	3	4	5	6	7	8	10
^a^Control vs ETem32	0.049	ns	ns	ns	Ns	0.0348	ns	0.0848
^a^Control vs ETem34	0.070	ns	ns	ns	Ns	ns	–	–
^a^ETem32 vs ETem34	ns	ns	ns	ns	Ns	ns	–	–
^a^Control vs (ETem32, ETem34)	0.022	ns	ns	ns	ns	0.051	–	–
^b^Control vs ETem32	ns	ns	ns	0.042	Ns	0.009	ns	0.0021
^b^Control vs ETem34	ns	ns	ns	ns	Ns	0.043	–	–
^b^ETem32 vs ETem34	ns	ns	ns	ns	Ns	ns	–	–
^b^Control vs (ETem32, ETem34)	ns	ns	ns	0.099	Ns	0.017	–	–

^a^ Significance of these contrasts was tested in analyses of variance carried out separately for each week using PROC GLM of SAS

^b^ Significance of these contrasts was tested in repeated measures analyses of variance carried out using PROC MIXED in SAS and testing using least squares means for each week separately

However, with the increase of swimming speeds during the 3^rd^ and 4^th^ weeks in all three treatments, treatment differences were not significant during this period. After the 4^th^ week, activity of tadpoles in ETem32 and ETem34 decreased up to the 7^th^ week until their death in ETem34. Activity of tadpoles in the control fluctuated after the 4^th^ week, with declines in the 5^th^ and 8^th^ weeks, but increases during the rest of the period up to the 10^th^ week. At the 7^th^ WAH, swimming speeds of tadpoles in the two elevated temperature treatments were significantly lower than in the control (Fig. 5 and Table 4). As metamorphosis of tadpoles in ETem32 was prolonged, their activity beyond the 7^th^ week showed fluctuations. At 10 WAH, swimming rate of tadpoles in ETem32 was significantly lower than in the control. Tadpole activity decreased substantially during the week prior to metamorphosis (in ECO2 and ETem32) or death (in ETem34) in elevated temperature and CO_2_ treatments. Interestingly, such a decline in activity was not observed prior to metamorphosis in the control. There were slight disagreements between the two methods of significance testing (i.e. PROC GLM and PROC MIXED) in the significance of some contrasts involving elevated treatments (Table 4). Hence, results of both methods are given in Table 4.

**Fig. 6:**
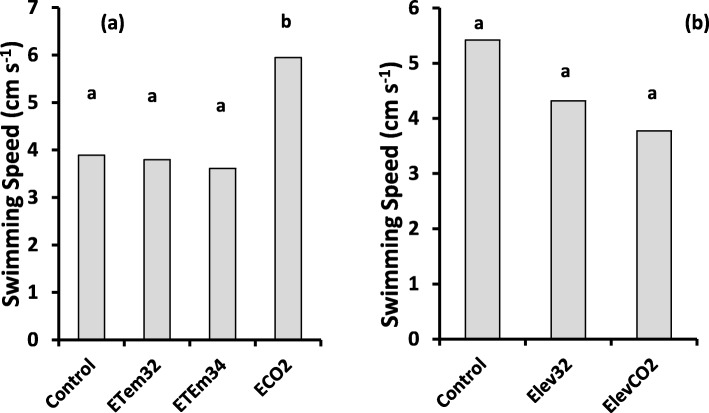
Effects of elevated temperature and CO_2_ treatments on swimming speed of tadpoles at Gosner stages 36–39 (**a**) and 42–46 (**b**). Control – Ambient CO_2_ (water pH = 7) and water temperature at 29 ± 1 °C; ETem32 – Water temperature elevated to 32 °C; ETem34 – Water temperature elevated to 34 °C. ECO2 – CO_2_ bubbled to water to maintain pH at 5.5–5.6. Each bar is the least squares mean of three replicate measurements. Least squares means with the same letter are not significantly different at *p* = 0.05

Comparison of activity of tadpoles at equivalent developmental stages showed significant variation among treatments at Gosner stage 36–39 (*p* = 0.032), but no significant variation at Gosner stage 42–46. At Gosner stage 36–39, swimming speeds of tadpoles in ECO2 were significantly greater than in other treatments which did not differ significantly among themselves (Fig. 6).

### Mortality of tadpoles

**Fig. 7:**
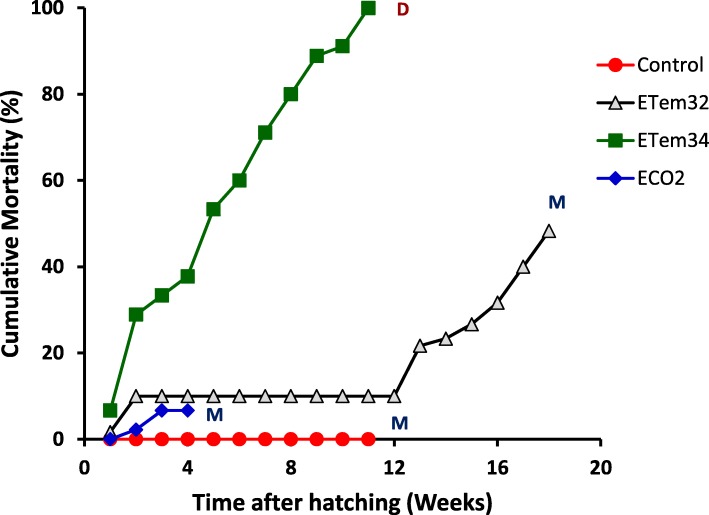
Progression of mortality of tadpoles in different treatments. Control – Ambient CO_2_ and water temperature at 29 ± 1 °C; ETem32 – Water temperature elevated to 32 °C; ETem34 – Water temperature elevated to 34 °C. ECO2 – CO_2_ bubbled to water to maintain pH at 5.5–5.6. M – Metamorphosis; D – Death. Red circles indicate the Control group. Grey triangles indicae the ETem32 group. Green squares indicated the ETem34 group. Blue diamonds indicate the ECO2 group

Cumulative mortality % (Cmort%) of tadpoles showed highly-significant variation among treatments throughout the experimental period (*p* < 0.0001) (Fig. 7). RM ANOVA showed highly-significant (*p* < 0.0001) variation in Cmort% with time, while the treatment x time interaction effect was also highly-significant. Tadpoles in the control treatment progressed through successive developmental stages to metamorphosis with 0% mortality. Across the whole experimental period, tadpoles in ETem34 showed significantly greater mortality than in all other treatments (Table 5). When the Cmort% data in different weeks were analyzed separately, a similar result was shown in all weeks except the first week (Additional file 3: Tables S3 and Additional file 4: Table S4). When the entire experimental period was considered in the RM ANOVA, tadpoles in ETem32 showed significantly greater mortality than those in the control (Table 5). Similarly, the two elevated temperature treatments, taken together, showed significantly greater mortality than the control. In contrast, Cmort% in ECO2 did not differ significantly from that in the control or ETem32, either across the first 4 weeks or in different weeks (Table 5 and Additional file 3: Table S3). However, Cmort% in ECO2 was significantly lower than that in ETem34, both overall and in different weeks.

**Table 5:** Significance of contrasts comparing the cumulative mortality percentages of tadpoles in different experimental treatments

Contrast	ECO2	ETem32	ETem34	(ETem32, ETem34)
Control vs	ns	0.0087	< 0.0001	< 0.0001
ECO2 vs	–	ns	< 0.0001	0.0022
ETem32 vs	–	–	< 0.0001	–

Significance of these contrasts was tested in repeated measures analyses of variance carried out using PROC MIXED in SAS for the whole experimental period

### Ammonia excretion

**Fig. 8:**
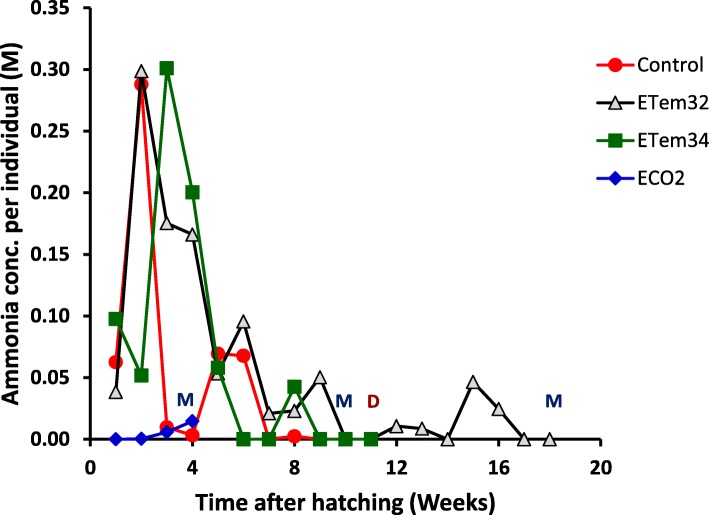
Time courses of variation of ammonia concentration in tank water in different treatments. Control – Ambient CO_2_ and water temperature at 29 ± 1 °C; ETem32 – Water temperature elevated to 32 °C; ETem34 – Water temperature elevated to 34 °C. ECO2 – CO_2_ bubbled to water to maintain pH at 5.5–5.6. M – Metamorphosis; D - Death

Tadpoles in ECO2 showed a continuous increase in ammonia excretion, measured as ammonia concentration (AmConc) in tank water, up to metamorphosis (Fig. 8). Other treatments showed substantial initial increases which were followed by decreases and further fluctuations of lesser amplitude. Across the whole experimental period, RM ANOVA showed a highly-significant (*p* = 0.0003) treatment x time interaction effect on AmConc. In addition, the main effects of treatments and time were also highly-significant (*p* < 0.0001). Significance testing of specific treatment contrasts by RM ANOVA showed that ammonia excretion of tadpoles in elevated temperatures were significantly greater than in the control (*p* = 0.0369) (Table 6). However, AmConc in the two elevated temperature treatments were not significantly different. When compared separately with the control, AmConc in ETemp32 was significantly greater (*p* = 0.0066) while that in ETemp34 was not significantly different. This was because of fluctuations in AmConc with time (Fig. 8). Therefore, the treatment differences varied at different times after hatching. For example at 2 WAH, AmConc in ETem32 was significantly greater than that in the control (*p* = 0.0114), but AmConc in ETem34 was lower (*p* < 0.0001) (Additional file 5: Table S5). However, during the following week, AmConc in the control and ETem32 decreased while that in ETem34 increased (Fig. 8) resulting in both elevated temperatures having greater AmConc than the control. Even though AmConc in the two elevated temperatures decreased at 4 WAH, they were still greater than that in the control. During the period from 5^th^ to 8^th^ WAH, there were no significant variations in AmConc between the elevated temperature treatments and the control. There was an increase in ammonia excretion in tadpoles in ETem32 at 9 WAH, thus raising its AmConc above those of the control and ETem34 (Fig. 8 and Additional file 5: Table S5).

**Table 6:** Significance of contrasts comparing the ammonia excretion of tadpoles in different experimental treatments

Contrast	ECO2	ETem32	ETem34	(ETem32, ETem34)
Control vs	ns	0.0066	ns	0.0369
ECO2 vs	–	0.0002	0.0054	0.0002
ETem32 vs	–	–	ns	–

Significance of these contrasts was tested in repeated measures analyses of variance carried out using PROC MIXED in SAS for the whole experimental period

Across the 5 weeks that tadpoles in ECO2 took to metamorphose, their ammonia excretion was not significantly different from those in the control (Table 6 and Fig. 8). However, AmConc in ECO2 was significantly lower than in both elevated temperature treatments. When the data from different weeks were analyzed separately, the above differences were most prominent during the second week (Additional file 6: Table S6). Here, AmConc in ECO2 was significantly lower than that in the control as well.

### Catalase enzyme activity

**Fig. 9:**
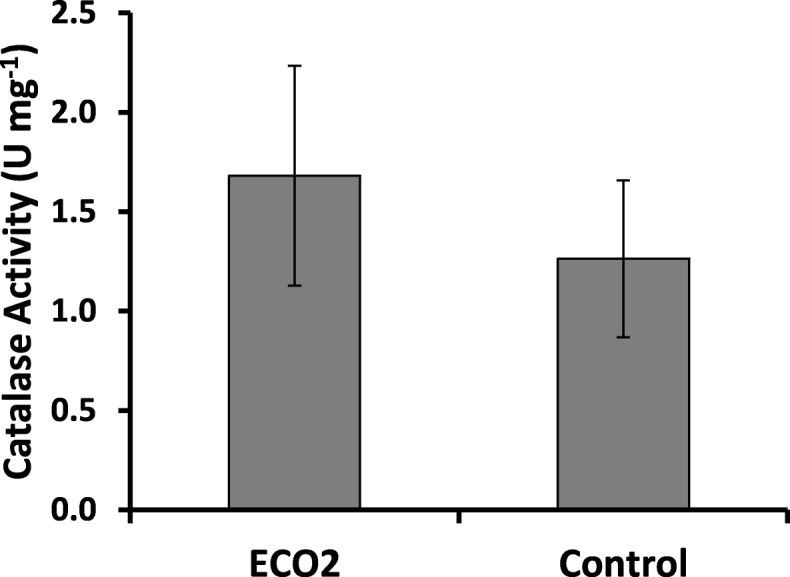
Mean catalase activity of tadpoles in ambient and elevated CO_2_ treatments. U mg^− 1^ – Units of catalase per mg of protein in the sample. Amount of catalase needed to degrade 1 μmol of H_2_O_2_ per min. is equivalent to one Unit. Control – Ambient CO_2_ and water temperature at 29 ± 1 °C; ECO2 – CO_2_ bubbled to water to maintain pH at 5.5–5.6. Error bars which are not overlapping indicate means are not significantly different at *p* = 0.05

Catalase activity of tadpoles in ECO2 was greater than in the control (Fig. 9). However, because of the greater variability among different replicates within each treatment, the above difference was not significant at *p* = 0.05. Measurement of catalase activity in the elevated temperature treatments was not possible because none of the individuals survived to reach the adult stage to extract the liver.

### Stomach lysozyme activity

**Fig. 10:**
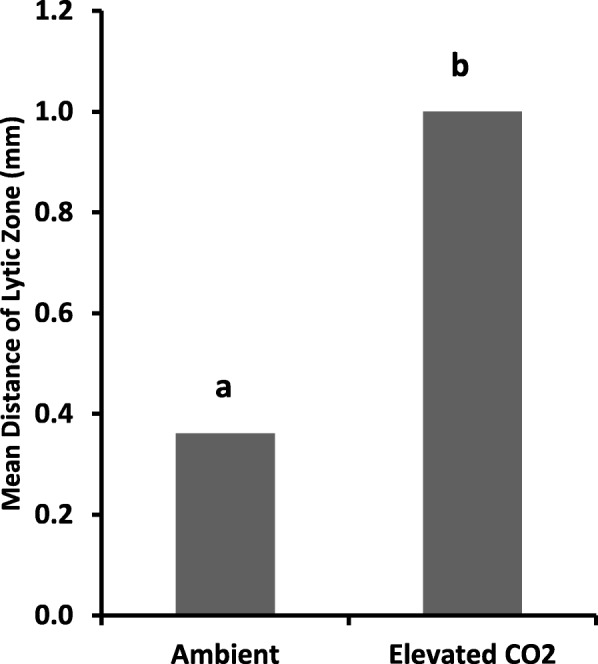
Mean distance of lytic zones of Ambient and Elevated CO_2_ treatments. Ambient – Ambient CO_2_ and water temperature at 29 ± 1 °C; ECO2 – CO_2_ bubbled to water to maintain pH at 5.5–5.6. Means with the same letter are not significantly different at *p* = 0.05

Determination of lysozyme activity of tadpoles in elevated temperature treatments was not possible because all individuals died before reaching Gosner Stage 36 in ETem34 while, in ETem32 individuals died within 24 h from reaching Gosner Stage 46. The distance of lytic zones resulting from lysozyme extracted from tadpoles of ECO2 was significantly higher than in the control (*p* = 0.046), thus indicating that elevated CO_2_ raised their lysozyme activity (Fig. 10 and Additional file 7: Plate S1).

### Haematological analysis

**Fig. 11:**
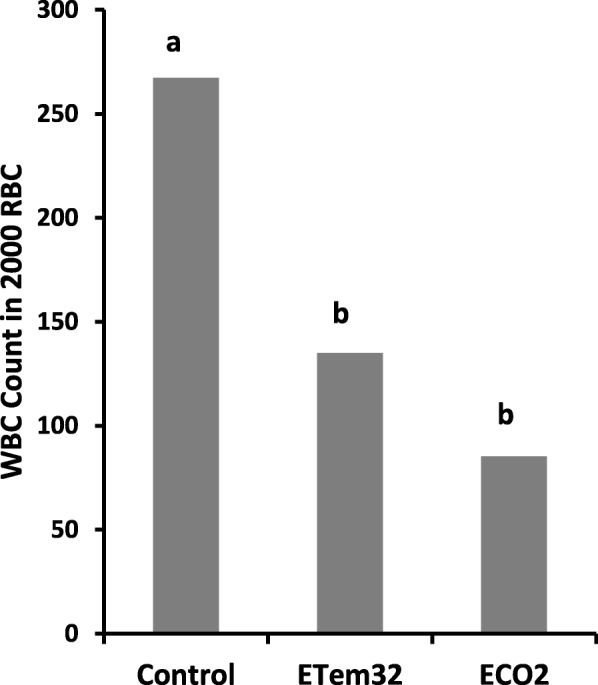
Counts of white blood cells (WBC) in 2000 red blood cells (RBC) in tadpoles under different treatments. Control – Ambient CO_2_ (water pH = 7) and water temperature at 29 ± 1 °C; ETem32 – Water temperature elevated to 32 °C; ECO2 – CO_2_ bubbled to water to maintain pH at 5.5–5.6. Means of WBC counts with the same letter are not significantly different at *p* = 0.05

Categorical data analysis showed that counts of white blood cells (expressed as a percentage out of 2000 red blood cells) varied significantly among treatments (*p* < 0.0001). Both elevated CO_2_ and elevated temperature at 32 °C reduced the WBC count significantly below that in the control (*p* < 0.0001) (Fig. 11). However, the frequency of WBCs in ETem32 did not differ significantly from that in ECO2 (*p* > 0.05). Haematological analysis of tadpoles in temperature elevated to 34 °C was not possible because none of the test animals survived until the Gosner Stage 46 at which blood was extracted.

**Plate 1:**
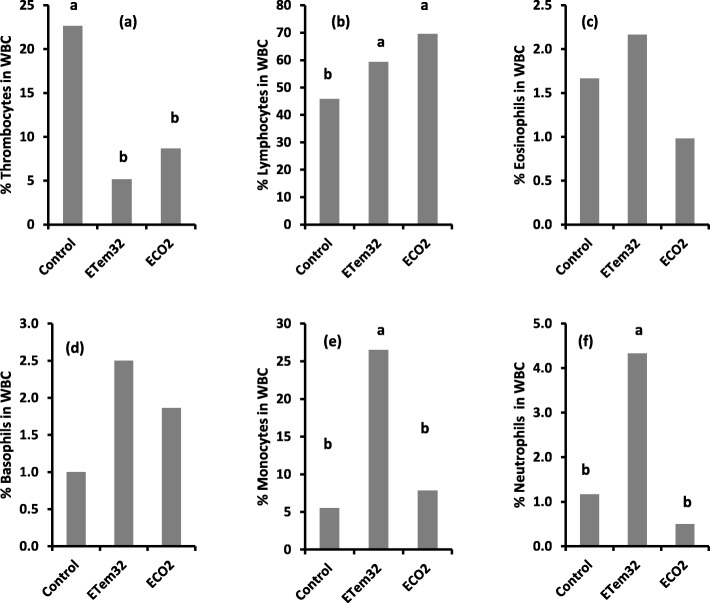
Percentages of different types of white blood cells such as (**a**) Thrombocytes, (**b**) Lymphocytes, (**c**) Eosinophils, (**d**) Basophils, (**e**) Monocytes, and (**d**) Neutrophils in tadpoles under different treatments. Control – Ambient CO_2_ (water pH = 7) and water temperature at 29 ± 1 °C; ETem32 – Water temperature elevated to 32 °C; ECO2 – CO_2_ bubbled to water to maintain pH at 5.5–5.6. Bars with the same letter are not significantly different at *p* = 0.05

Different types of WBCs observed in the blood stains included neutrophils, monocytes, basophils, eosinophils, thrombocytes, and lymphocytes (Plate 1). Significant (*p* < 0.01) variation among treatments was observed in the percentages of thrombocytes, lymphocytes, monocytes, and neutrophils in WBCs (Table 7).

**Table 7:** Significance of treatment effects on percentages of white blood cells and its different types

Cell type	All treatments	Treatment comparison		
		Control vs ETem32	Control vs ECO2	ETem32 vs ECO2
% WBC in RBC	< 0.0001	< 0.0001	< 0.0001	ns
% Thrombocytes	0.0006	< 0.0001	0.0154	ns
% Lymphocytes	0.0007	0.0262	0.0004	ns
% Eosinophils	ns	–	–	–
% Basophils	ns	–	–	–
% Monocytes	< 0.0001	< 0.0001	ns	< 0.0001
% Neutrophils	0.0046	< 0.0001	ns	< 0.0001

Significance of treatment effects and treatment comparisons were tested using a Z-statistic calculated from the maximum likelihood frequencies and their standard errors obtained in a categorical data analysis using PROC CATMOD in SAS

**Fig. 12:**
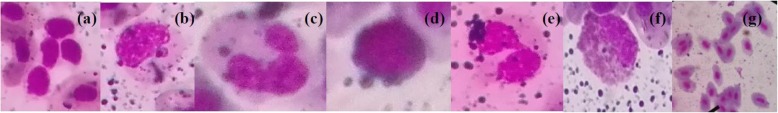
Blood cells observed in the liver tissue of tadpoles: (**a**) Thrombocytes, (**b**) Monocyte, (**c**) Neutrophil, (**d**) Lymphocyte, (**e**) Eosinophil, (**f**) Basophil, and (**g**) Erythrocytes

In contrast, percentages of eosinophils and basophils did not differ significantly among treatments. Elevated temperature (ETem32) and CO_2_ (ECO2) reduced the percentage of thrombocytes (Fig. 12a) and increased the percentage of lymphocytes (Fig. 12b). However, there was no significant difference between ETem32 and ECO2 in the percentages of thrombocytes and lymphocytes. In contrast, ETem32 significantly increased the percentages of monocytes (Fig. 12e) and neutrophils (Fig. 12f), whereas ECO2 did not cause a significant change.

### Deformities

**Plate 2:**
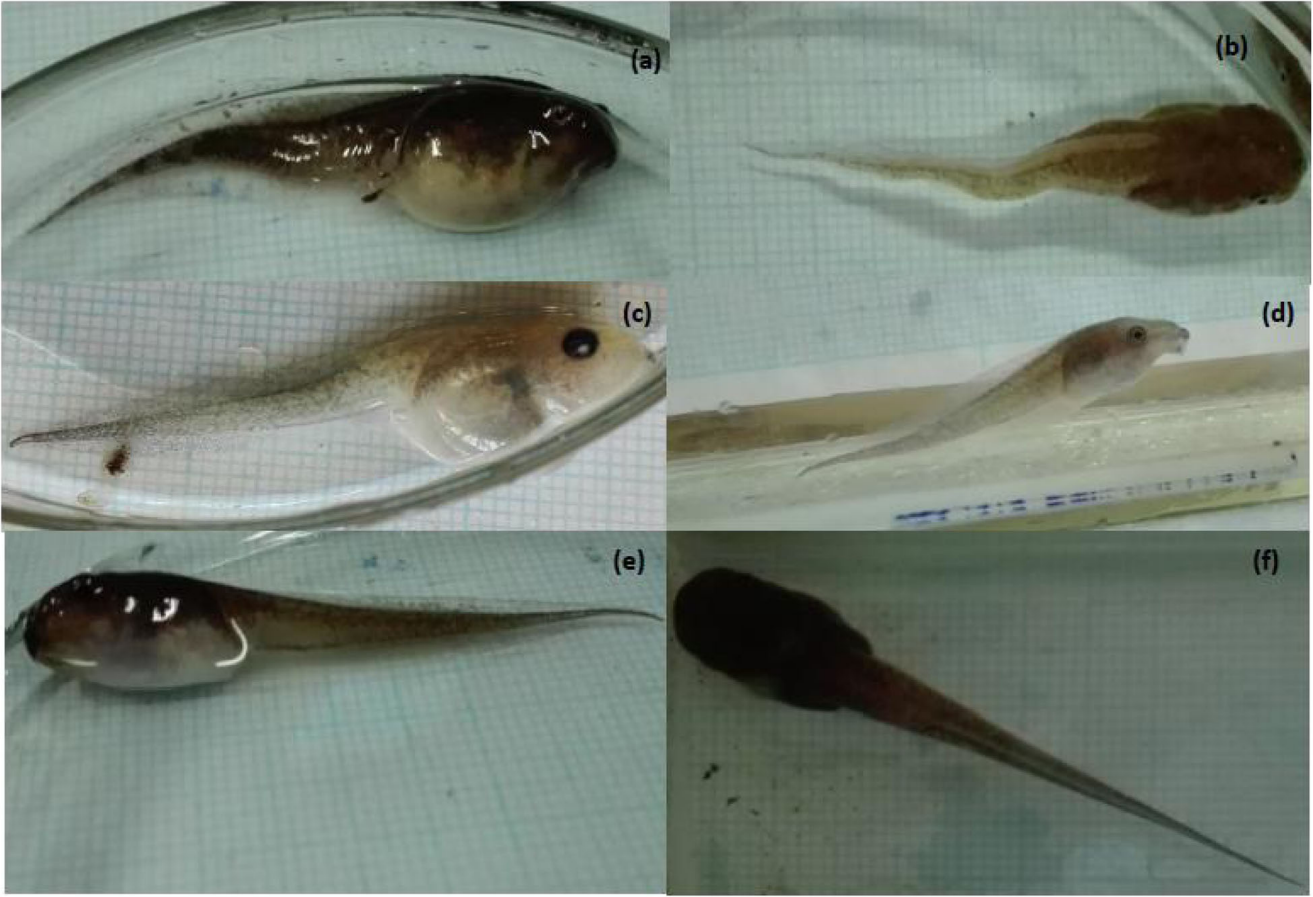
Tadpoles with (**a**) Oedema, (**b**) Tail kink, (**c**) Pale pigmentation, (**d**) Beaked snout and (**e**) and (**f**) Normal tadpoles

In the control treatment, the only deformities observed were tail kinks and pale pigmentation while deformities such as tail kinks, oedema, beaked snout, and pale pigmentation were observed in ETem32 (Plate 2). In ECO2, the only deformity observed was the beaked snout. The highest percentage of deformities was observed in ETem32 (Table 8). Deformities could not be identified in ETem34 as all test animals died at an early stage.

**Table 8:** ^a^Percentage prevalence of deformities among test animals in different experimental treatments

Deformity	Control	Elevated CO_2_	Elevated temperature at 32 ± 0.5 °C
Oedema	–	–	17.1
Tail kink	8.9	–	14.6
Pale pigmentation	6.7	–	26.8
Beaked snout	–	11.9	29.3

^a^Percentage of animals showing deformities from among test animals in each treatment

## Discussion

### Experimental treatments viz-à-viz the thermal and climate change reality of *P. cruciger*

The control treatment of our experiment represents the current thermal regime experienced by *P. cruciger* in Sri Lanka. Temperature in the control treatment (29 °C) is typical of the lower altitude, humid tropical climate in South-Western Sri Lanka where the experiment was carried out. As Sri Lanka is located at a latitudinal range of 6-11^o^N, it experiences a relatively-narrow diurnal variation in ambient temperature. In this climatic zone, the mean daytime maximum and the mean night-time minimum are ca. 31^o^ and 27 °C respectively. The day length is around 12 h throughout the year with an amplitude of only 40 min between the longest and the shortest day of the year. Therefore, 29 °C in the control treatment represents a mean temperature that tadpoles of *P. cruciger* would actually experience in their natural habitats in Sri Lanka. The two elevated temperatures, viz. 32^o^ and 34 °C, are based respectively on projected temperature increases of 3^o^ and 5 °C by mid-twenty-first Century according to the ‘best-case’ (RCP2.6) and ‘business-as-usual’ (RCP8.5) scenarios of the IPCC [1, 2]. We acknowledge that the use of constant temperatures in our experiment, rather than a diurnal variation around the mean temperatures, represents a deviation from the actual thermal regimes that tadpoles experience in their natural habitat. However, we believe that this deviation would not introduce a significant deviation in the observed response of tadpoles because of: (a) the relatively-narrow diurnal variation of temperature in this climate; and (b) the long-term decreasing trend in the diurnal temperature range due to the night-time minimum temperature increasing faster than the daytime maximum temperature [75–77]. Therefore, the currently narrow diurnal temperature range in Sri Lanka is likely to narrow down further in the future.

The elevated CO_2_ treatment in our experiment was imposed based on the projected pH reduction in the future climate change scenarios [1, 2]. Here, the absence of ambient air bubbling in the ECO2 treatment could have caused a difference in oxygen concentrations between ECO2 and the rest of the treatments. However, we believe that ECO2 was receiving adequate oxygen via natural dissolution so that any difference in oxygen concentration in tank water did not introduce a significant deviation in the organism responses.

### Growth, development and survival: effects of low pH caused by elevated CO_2_

Our results demonstrate that reduced pH due to increased dissolved CO_2_ and increased water temperature cause significant changes in the rates of development and growth of *Polypedates cruciger* tadpoles. These provide important indicators of how future climate change may influence anuran amphibians. While consequent reduction of water pH accelerated the progression of tadpoles through successive developmental stages, elevated temperature delayed their development (Fig. 1). Our observations on the impact of elevated CO_2_ contradict those of previous studies, where exposure to lower pH resulted in longer larval periods [78–80]. This is not surprising as time to metamorphosis shows phenotypic plasticity, particularly in response to environmental stressors, to ensure the survival of young adults [81]. Furthermore, environmental stressors could influence the hormonal control of metamorphosis [82]. Accordingly, lower pH in the elevated CO_2_ treatment could activate the hypothalamus-pituitary-interrenal axis and accelerate metamorphosis by stimulating the secretion of thyroid and glucocorticoid hormones. Environmental stressors are known to result in fluctuations in thyroid hormone levels which affect metamorphic, developmental and morphometric traits of anuran tadpoles [83, 84]. Traits such as shorter larval period, reduced morphometrics and reduced survival which negatively affect organisms are driven by disruptions in the thyroid hormone homeostasis caused by environmental stressors. Increased thyroid hormone levels resulting from environmental stressors can lead to undersized, early metamorphosed anuran tadpoles [83, 85, 86]. This is in agreement with the shortened larval stages and lower morphometrics observed in tadpoles raised under elevated CO_2_ conditions in our study. Hence, disruption in thyroid hormone levels due to acid stress could be a possible explanation for this observation. Future studies on thyroid hormone levels of tadpoles of *P. cruciger* exposed to elevated CO_2_ levels would be useful for confirmation of this possibility.

Possible hypoxic conditions in the elevated CO_2_ treatment due to the absence of air bubbling may also have contributed to the changes in the hormonal control induced by lower pH. Furthermore, elevated CO_2_ and lower pH increased both developmental and growth rates, measured in terms of their morphometrics, (Fig. 2 and Additional file 2: Table S2). This contrasts with the findings of Freda and Dunson [87] and Böhmer and Rahmann who reported reduced growth rates due to acid stress at the embryonic and early larval stages [88]. One possible explanation of this disagreement is the differential responses among different amphibian species as the previous work has been done on a different frog species (i.e. *Rana temporaria*). On the other hand, our observation could be an acquired adaptation of *P. cruciger* to the acidic nature of soils in the wet zone of Sri Lanka [89], as supported by the work of Silva and Munaweera [90]. It has been shown that water in the egg collection region (i.e. Kandy) has lower pH, which is within the pH range used study. Our observation of shorter larval periods and earlier maturity could be an adaptive measure for chronic exposure to low pH levels. In nature, tadpoles mostly occupy temporary ponds that are highly vulnerable to drying followed by high acidity. In such a situation, earlier maturity allows a greater chance of survival and constitutes adaptive plasticity [91–94]. Furthermore, by shortening the larval period under a stressed condition, the risk of predation [95], desiccation [96] and infection [97] is reduced. Therefore, our observation of accelerated development while maintaining growth rates of *P. cruciger* tadpoles in response to chronic exposure to elevated CO_2_ and increased acidity may indicate an adaptive response to environmental change in their natural habitat.

It should be noted that in freshwater aquatic environments, decomposing organic matter constitutes an additional source of CO_2_. Therefore, in a future climate, pH of freshwater could decrease below the level predicted solely based on elevated atmospheric CO_2_. Thus, it is likely that freshwater organisms may be subjected to even lower levels of acidity than those in the present experiment. Therefore, adaptive responses as observed in our work could be of greater significance. Furthermore, it is possible that CO_2_ generated from additional sources such as decomposing organic matter could show seasonal variation depending on climatic (e.g. precipitation, temperature) and vegetation (e.g. litter fall) parameters. Accordingly, ability to adapt to fluctuating pH levels may also become an important trait for future survival of organisms which spend at least part of their life cycle in aquatic environments.

Even though reduced water pH did not affect early growth rates of tadpoles, shorter durations taken for progression through successive developmental stages (i.e. Gosner Stages 36–39 and 42–46) meant that at equivalent developmental stages, their morphometrics were lower than those in the control (Figs. 3 and 4). This is in accordance with previous studies [78, 80], which have shown that tadpoles exposed to low pH were lower in body size at metamorphosis in comparison to those at ambient pH. These metamorphic traits (i.e. size and timing of metamorphosis) are critical factors of fitness [80]. When an individual is smaller at the age of metamorphosis, it may have a lower chance of survival and reduced reproductive success [92, 94, 98]. This can be a contributory factor to the decline of amphibian populations globally as increasing CO_2_ reduces pH in their habitats.

Reduced growth of tadpoles in acidic conditions could be due to disrupted sodium balance [80] as even moderately acidic water disrupts the ion-regulatory process of larval amphibians [99]. Furthermore, living in a pH stressed condition requires maintenance of homeostasis while being subjected to changes of biochemical, physiological, and molecular processes [100]. This requires a greater energy expenditure, which could also result in decreased body size at metamorphosis.

Comparison of the observed mortalities of tadpoles in the present work (Fig. 7) with those in previous work is complicated by the fact that mortality and survival rates are highly dependent on species [87, 99]. Lower mortality of tadpoles exposed to acidic pH (in comparison to higher mortality levels in elevated temperature treatments) in our study agrees with observations on salamanders [101], but contrasts with 25% higher mortality of *Rana temporaria* tadpoles raised in acidic pH [80]. Generally, pH levels lower than 4.5 have a lethal effect on amphibian larvae while pH levels lower than 5 cause hatching and growth inhibition [102]. On the other hand, pH levels above 5 are considered sub-lethal with hatching proceeding uninhibited, but with detrimental effects on metabolism. This agrees with our observations as the pH range of current study (i.e. 5.5–5.6) falls within the sub-lethal range. In accordance with previous studies [80], mortalities in low pH treatment were not observed until commencement of metamorphosis (Fig. 7). Physiological stress that tadpoles experience with the onset of metamorphosis could have caused the observed increase in mortality with approaching metamorphosis.

### Growth, development and survival: effects of elevated temperatures

Our observations demonstrate that the two elevated temperature treatments (i.e. 32 °C and 34 °C), corresponding to ‘best-case’ (RCP2.6) and the ‘worst-case’ (RCP8.5) scenarios of future climate change [1, 2], had substantial adverse impacts on the growth, development, and survival of *P. cruciger* tadpoles. Their growth and development show a high degree of sensitivity to the 4 – 5 °C increase in temperature in the present study. In contrast to the response to elevated CO_2_, elevated temperatures delayed development (Fig. 1) and reduced growth rates (Fig. 2 and Additional file 2: Table S2). Death before metamorphosis at 34 °C shows that the lethal temperature for tadpoles of *P. cruciger* lies between 32 °C and 34 °C. This shows that even though *P. cruciger*, being a species endemic and evolved in a tropical climate with a relatively high temperature regime, is adapted to inhabit a higher temperature (e.g. 29 °C in the present situation) than temperate species such as *Rana temporaria* and *Bufo bufo* [103], it has a narrow thermal tolerance range. This is in accordance with the postulation of Janzen [56] that organisms that inhabit the relatively less variable thermal regime of tropical climates are acclimated and evolutionary adapted to a narrower fluctuation in their environment than comparable organisms inhabiting the more variable temperate climates. Evidence supporting the applicability of Janzen’s postulation to anuran amphibians has been shown by Ruthsatz et al. [83] who found that larvae of the African clawed frog (*Xenopus laevis*) reared at higher temperatures had a lower range of thermal tolerance that those reared at lower temperatures, despite having a higher maximum thermal limit. Similarly, Drakulic et al. [104] observed that morphometrics, physiological condition and activity of metamorphs of *Rana temporaria* populations originating from warmer habitats show adaptation to warmer temperatures than metamorphs originating from cooler habitats. Adaptation to the local environment and its fluctuations is particularly important for tadpoles of anuran because of their limited capability for thermoregulation and movement towards favourable habitats [105]. However, data on local adaptations and tolerance limits of *P. cruciger* and/or similar local species are lacking. Hence, we suggest this as a future direction of study. Increasing mortality rate of tadpoles exposed to 34 °C from the beginning of the experiment indicated their inability to acclimate to the increased temperature (Fig. 7). The time at which 100% mortality was observed in this treatment was approximately synchronous with the time at which tadpoles in the control treatment initiated metamorphosis. Physiological stress caused with the onset of metamorphosis, exacerbated by the elevated temperature, could also have contributed to tadpole mortality before metamorphosis at 34 °C. This indicates that tadpoles of *P. cruciger* have little chance of acclimation and survival in the predicted future temperature increases under the ‘business-as-usual’ scenario (RCP 8.5).

On the other hand, tadpoles exposed to elevated temperature at 32 °C showed only 10% mortality during the first 2 weeks (Fig. 7). It remained constant until commencement of metamorphosis indicating the ability of tadpoles of *P. cruciger* to tolerate to 32 °C. However, this was possible only for the larval stage as mortality was observed in all late-stage larvae (Gosner Stage 42–46) raised at 32 °C within 24 h of metamorphosis. This is in agreement with a similar study on a montane frog species *Eleutherodactylus portoricensis* which rarely experienced temperatures above 30 °C [106]. Similarly, *P. cruciger* rarely experiences temperatures as high as 32 °C because of the altitude (300–1525 m) of its natural habitat. Hence the late-stage larvae of *P. cruciger* may not have developed sufficient adaptations to tolerate dehydration and other physiological changes caused by temperatures above 29 °C. This could be the reason for its mortality at the exposure to elevated temperature. However, survival of tadpoles at 32 °C up to the stage of metamorphosis indicates the possibility of this species developing adaptations for survival under predicted future temperatures in the best-case scenario (RCP2.6). This is supported by the observation of Ruthsatz et al. [83] that tadpoles developed at warmer temperatures show higher maximum thermal limits. Drakulic et al. [105] also provide evidence that anuran amphibians have the capacity to adapt to warmer temperatures after they have experienced a higher temperature for some period.

Growth rates of tadpoles exposed to 32 °C and 34 °C decreased relative to that of tadpoles in ambient temperature from the 4th week onwards (Fig. 2). This is because growth and development would proceed slowly when temperature is supra-optimal relative to the range required by the cellular processes [107]. Tadpoles raised at 32 °C have taken a longer time than tadpoles at ambient temperature to reach the stage of metamorphosis, which is in accordance with similar studies [107]. Reduced morphometrics and delayed metamorphosis could be due to changes in energy allocation with most of it being diverted to maintain a high routine metabolic rate. Furthermore, reduced morphometric growth leads to delays in attaining the minimum size threshold required for metamorphosis [83, 108–110]. Reduced morphometrics and longer larval period of tadpoles exposed to 32 °C makes *P. cruciger* mostly vulnerable to predation, infection and desiccation in future temperature predicted even under the best-case-scenario (RCP 2.6).

Negative traits such as the reduced morphometrics and reduced survival observed in both elevated temperature and elevated CO_2_ conditions could have yielded from changes of the environmental stressor-induced thyroid hormone levels [83, 84]. Existing studies show that thyroid hormone levels altered due to environmental stressors have resulted in substantially low survival rates [83]. Therefore, we recommend that future studies are carried out on *P. cruciger* to confirm how thyroid hormone homeostasis influence reduced survival and lower morphometrics observed under elevated CO_2_ and temperature.

#### Activity of tadpoles

Elevated CO_2_ and temperature caused changes in the activity of tadpoles. Elevated CO_2_ increased swimming speed of tadpoles during their early growth stages (i.e. Gosner stages 26–30), elevated temperatures reduced tadpole activity than that of the control (Fig. 5). This observation agrees with previous studies which demonstrate that high temperatures impair motility of tadpoles [107]. Therefore, future temperature increases could increase vulnerability of *P. cruciger* tadpoles for predation due to reduced motility. We suggest further studies with predatory pressure incorporated to investigate this aspect. The marked reduction of swimming speed that was observed in the week prior to metamorphosis in elevated CO_2_ and elevated temperature treatments could be due to reduced metabolism and activity during metamorphosis. However, stress induced by reduced pH and increased temperature could also have contributed to this reduction as it was not observed in the control treatment.

### Catalase enzyme activity

Increased catalase enzyme activity in tadpoles raised in low pH in our work is in agreement with similar studies [111]. Amphibians live in small, ephemeral aquatic environments are highly susceptible to large fluctuations of pH and temperature, where the resulting biochemical, physiological and molecular changes require maintenance of homeostasis and a higher rate of metabolism. This increases respiration and increases production of reactive oxygen species (ROS) as a byproduct, leading to oxidative stress. This could increase the activity of enzymes such as catalase which is a key component of antioxidant defence systems [100, 112].

### Ammonia excretion

Concentration of released ammonia is an indication of excretory metabolism of tadpoles. In our study, elevated CO_2_ decreased ammonia excretion of tadpoles (Fig. 8, Table 6 and Additional file 6: Table S6), which contradicts observations on anuran tadpoles and fish exposed to acidity [99, 113–115]. One explanation for this discrepancy may be the differences between species and the rates of exposure to low pH. In an acidic medium, more ammonia would be protonated upon excretion as a result of increased external H^+^ ion concentration. It could affect the overall ammonia excretion [116–118]. The increase in ammonia gradient across gills and skin would facilitate greater ammonia excretion. Although acute exposure may give this result, as animals in this study were chronically exposed to low pH, a new equilibrium is likely to establish at lower blood ammonia, resulting in reduced concentrations of excreted ammonia [99]. Furthermore, moderately acidic water disrupts the ion-regulatory process of larval amphibians. Short- and long-term exposure to low pH resulted in amphibians losing 21–62% of body sodium resulting in diffusive loss of ions [87]. This was mostly due to stimulation of sodium efflux. Therefore, a part of the increase in ammonia excretion in previous studies may be due to passive ammonia efflux across a leaky gill or membrane. Decrease of ammonia excretion in our study could be due to the sodium present in the citrate buffer in the medium preventing the increased sodium efflux resulting from low pH. Nevertheless, reduced ammonia excretion in the elevated CO_2_ (i.e. lower pH) treatment merits further investigation.

In contrast to elevated CO_2_, elevated temperatures increased ammonia excretion in tadpoles (Fig. 8, Table 6 and Additional file 5: Table S5). This could be due to heat stress induced faster metabolism and consequently higher respiration rates. Protein catabolism is increased with increased respiration and thereby increases the excretion of ammonia nitrogen. Weekly variation in ammonia excretion showed reductions during the weeks that a majority of tadpoles were undergoing metamorphosis (e.g. week 7 onwards in the control, week 9 onwards in elevated 32 °C). This was probably because of reduced metabolism and activity during metamorphosis. Reduced ammonia excretion as metamorphosis approached could have been due to tadpoles changing their excretory metabolism from ammonia to urea. However, we do not have information on whether this transition occurred and if so when in this experiment.

### Immunity

Research on the impact of elevated temperature and low pH on the immune response of tadpoles is limited, with most reporting the impact of reduced temperatures (i.e. winter, hibernation) on the immunity of amphibians. Reduction in the white blood cell (WBC) count in the elevated CO_2_ treatment (Fig. 11) is in agreement with previous studies where a reduction of splenic WBC was observed in *Rana pipens* tadpoles exposed to pH 5.5 [45]. Increased lymphocyte (Fig. 12b), monocyte (Fig. 12e) and neutrophil (Fig. 12f) counts in tadpoles raised in 32 °C suggest an infection as they are the major WBCs involved in phagocytosis. This is supported by the observed deformities which could possibly be related to trematode infections and increased mortality of adults [62, 119]. Reduction of thrombocytes in tadpoles experiencing elevated temperature and reduced pH (Fig. 12a) indicates reduced immunity as thrombocytes play an important role in hemostasis by plugging damaged blood vessels.

Significantly larger lysis zone in the low pH treatment (Fig. 10) indicated increased lysozyme activity in tadpoles compared to those in ambient pH. This may suggest an infection in tadpoles in this treatment [44]. These observations suggest that environmental fluctuations such as elevation of temperature and pH make tadpoles of *P. cruciger* more vulnerable to infections, even under the best-case scenario of climate change.

### Deformities

Developmental and morphological deformities such as oedema, beaked snout, pale pigmentation, and tail kink were observed (Plate 2). Out of these, only two were observed in tadpoles raised under ambient conditions and those were also in very low percentages. Beaked snout was observed in tadpoles raised in low pH while all deformities were observed in high percentages in tadpoles raised in 32 °C. These deformities usually result from filling of fluid in the body cavity (oedema), pale-colored liver, congestive and hemorrhagic organs, malformations in melanin producing cells, and malformations of the spinal cord [120]. Also, most of these deformities were similar to those induced by trematode infections [62, 119], which indicates an infection in tadpoles exposed to 32 °C, where most deformities were observed. Deformities could also be caused by disruption of thyroid hormone system [120], which is highly-sensitive to environmental stressors such as higher temperatures [83] and toxicants in the environment such as air pollutants and agro-chemicals [85].

These malformations hamper movements of tadpoles and make them vulnerable [119], such as pale pigmentation makes them more vulnerable to predation. This is applicable in the present study because tadpoles of *P. cruciger* normally develop in temporary, shallow ponds formed on places such as rock crevices and garden ponds which are usually dark in background. Therefore, having a pale pigmentation in such a background can make the tadpoles more conspicuous for predators, thus increasing their vulnerability. On the other hand, motor disorders (tail kink and oedema) and mouth deformities (beaked mouth) impair swimming, (by loss of balance) foraging and feeding [48]. This could have contributed to the reduced growth rate and eventually the reduced survival in tadpoles raised in 32 °C. Thus, it is clear that elevated temperatures and low pH levels, even under the best-case scenario, induce deformities in *P. cruciger* tadpoles, which affect them negatively by increasing their susceptibility to predation and reducing foraging success in natural habitats. This observation carries significant weight, although the exact reason behind increased occurrence of deformities under elevated temperature and low pH levels cannot be pinpointed. That is because, to the best of our knowledge, this is the first time that occurrence of deformities in *P. cruciger* has been discussed in the context of climate change. Existing literature has discussed how toxicity of pesticides induces deformities in this species [62, 85, 119]. However, the influence of increased acidity and elevated temperature on occurrence of deformities has not been discussed with respect to this species. Furthermore, there exists a remarkable paucity of research discussing the relationship between elevated temperatures and deformities in anurans, although studies exist on how these elevated acidic conditions induce deformities in other anuran species [121, 122]. Therefore, species-specific research, designed to identify the exact causes behind increased prevalence of deformities in tadpoles reared under elevated temperature and acidic conditions is recommended.

### Future directions

To the best of our knowledge, this is the first time that impact of climate change on this species has been assessed, although the impacts of agrochemical toxicity on *P. cruciger* have been investigated. That makes the findings of this study significant and novel, opening several paths for future research based on detailed seasonal field studies, assessing the impact of climate change on this species. An endemic species such as *P. cruciger* usually experience constant conditions close to its physiological optima, since Sri Lanka is a tropical country not experiencing annual seasonal variations [55–57]. Therefore it is likely for *P. cruciger* to have evolved very limited adaptations to tolerate fluctuating environmental conditions. That makes this species more vulnerable to climate change [58, 59]. Therefore, assessing genetic aspects determining the tolerance capacity and physiological responses of *P. cruciger* to future climatic conditions and empirical studies based on computational modeling predicting the fate of this species in future climatic conditions are highly recommended as future research avenues.

Furthermore, *P. cruciger* is a species prevalent in both ‘wet’ (i.e. humid tropical) and ‘dry’ (i.e. sub-humid tropical) zones of Sri Lanka, across an altitude ranging up to 1525 m above sea level, in a wide and patchy distribution pattern. Physiological and behavioral responses of anurans to environmental stressors such as thermal stress are highly population-specific [104, 105, 123]. That is because populations located in different habitats are likely to have developed variable local adaptations resulting in a discrepancy in the ways that they respond to environmental stressors. Hence, there is a possibility that different populations of *P. cruciger* distributed in varying climatic zones and altitudes of the island to show different responses to the factors tested in this study. Findings of this study are based on test animals that were collected only from the wet zone. We recommend future studies to be conducted on test animals collected from a wider environmental range to obtain a more generalized insight of how *P. cruciger* would respond to climate change.

## Conclusions

This work provides a comprehensive analysis of the physiological response of *Polypedates cruciger* (Common hourglass tree frog) to two key aspects of climate change; increased temperature and increased CO_2_-induced reduced pH in aquatic habitats. Based on the observed results, we conclude that chronic exposure to elevated temperature and CO_2_ alters many physiological responses of tadpoles of *P. cruciger* which could increase their susceptibility to predation, infection, desiccation, and mortality. Elevated temperature and low pH-induced effects incorporate reduced morphometrics and body size, increased motility changes in immune cells and deformities. Hence, we conclude that climate change can possibly impose a significant threat to *P. cruciger*. However, it may be possible for the species to adapt to low pH conditions by accelerating development and shortening the larval period. Increased activity of antioxidant enzymes such as catalase and an immune response via increased production of white blood cells could constitute further adaptive responses to physiological stress induced by altered temperatures and pH. This preliminary study is the first of its kind to report the physiological responses of an endemic amphibian species of Sri Lanka to future climate change. We acknowledge that the constant temperatures used in our experimental tanks deviate from the fluctuating temperatures in a natural habitat. However, in view of the narrow amplitude of the diurnal variation of temperature in tropical climates, our findings provide important insights into the response of tropical anuran amphibians to future climate change.

## Supplementary information

**Additional file 1: Table S1.** Second-order polynomial functions fitted to variation of tadpole morphometrics with time (wk).

**Additional file 2: Table S2.** Growth rates of tadpole morphometric characters at different developmental stages.

**Additional file 3: Table S3.** Significance of contrasts comparing cumulative tadpole mortality in elevated CO_2_ with that of other treatments.

**Additional file 4: Table S4.** Significance of contrasts comparing cumulative tadpole mortality in elevated temperatures with that of others.

**Additional file 5: Table S5.** Significance of contrasts comparing ammonia excretion of tadpoles in elevated temperatures with that of others.

**Additional file 6: Table S6.** Significance of contrasts comparing tadpole ammonia excretion in elevated CO_2_ with that of other treatments.

**Additional file 7: Plate S1.** Lytics zones on gel plates 48 h after innoculation. Con - Ambient CO_2_ (water pH = 7) and water temperature at 29 ± 1 °C; ECO2 – CO_2_ bubbled to water to maintain pH at 5.5–5.6. R1 – R4 – Replicates.

## Data Availability

The data sets generated in the current study are available from the corresponding author on reasonable request.

## Abbreviations

AmConc: Ammonia concentration in tank water
C_a_: Atmospheric CO_2_ concentration
Cmort%: Cumulative percentage of mortality
ECO2: Elevated CO_2_ treatment
ETem32: Treatment in which tank water temperature was elevated to 32 °C
ETem34: Water temperature elevated to 34 °C
IPCC: Intergovernmental Panel on Climate Change
RCP: Representative Concentration Pathway
ROS: Reactive oxygen species
WAH: Weeks after hatching
WBC: White blood cells
