# Supplementary material for: The impact of elevated temperature and CO2 on growth, physiological and immune responses of Polypedates cruciger (common hourglass tree frog)
Source: Front Zool. 2020 Jan 13;17:3. doi: 10.1186/s12983-019-0348-3 (PMC6958743; doi:10.1186/s12983-019-0348-3)
Supplement: Supplementary file 1 — Additional file 1: Table S1. Second-order polynomial functions fitted to variation of tadpole morphometrics with time (wk). [file 12983_2019_348_MOESM1_ESM.docx]

**Table S1.** Second-order polynomial functions fitted to variation of tadpole morphometrics with time (wk)

| Morphometric character | Treatment | Function | R^2^ |
| --- | --- | --- | --- |
| Total body length, TBL, (cm) | Control | TBL = -0.031wk^2^ + 0.774wk + 0.771 | 0.968 |
|  | ETem32 | TBL = -0.018wk^2^ + 0.587wk + 0.841 | 0.956 |
|  | ETem34 | TBL = -0.065wk^2^ + 0.972wk + 0.351 | 0.985 |
|  | ECO2 | TBL = -0.128wk^2^ + 1.494wk - 0.069 | 0.938 |
|  |  |  |  |
| Snout-vent length (SVL), (cm) | Control | SVL = -0.007wk^2^ + 0.195wk + 0.352 | 0.916 |
|  | ETem32 | SVL = -0.006wk^2^ + 0.174wk + 0.325 | 0.952 |
|  | ETem34 | SVL = -0.014wk^2^ + 0.225wk + 0.187 | 0.966 |
|  | ECO2 | SVL = -0.050wk^2^ + 0.510wk - 0.011 | 0.903 |
|  |  |  |  |
| Tail length (TL), (cm) | Control | TL = -0.025wk^2^ + 0.579wk + 0.364 | 0.965 |
|  | ETem32 | TL = -0.011wk^2^ + 0.391wk + 0.569 | 0.934 |
|  | ETem34 | TL = -0.048wk^2^ + 0.728wk + 0.029 | 0.974 |
|  | ECO2 | TL = -0.110wk^2^ + 1.206wk - 0.444 | 0.943 |
|  |  |  |  |
| Body width (BW) (cm) | Control | BW = -0.003wk^2^ + 0.097wk + 0.158 | 0.986 |
|  | ETem32 | BW = -0.003wk^2^ + 0.095wk + 0.127 | 0.962 |
|  | ETem34 | BW = -0.006wk^2^ + 0.107wk + 0.117 | 0.987 |
|  | ECO2 | BW = -0.011wk^2^ + 0.118wk - 0.225 | 0.681 |

**Note:** Time is measured in terms of weeks (wk) after hatching. Control – Ambient CO_2_ (water pH=7) and water temperature at 29 ± 1^o^C; ETem32 – Water temperature elevated to 32 ± 0.5^o^C; ETem34 – Water temperature elevated to 34 ± 0.5^o^C. ECO2 – CO_2_ bubbled to water to maintain pH at 5.5-5.6.
