# Supplementary material for: The impact of elevated temperature and CO2 on growth, physiological and immune responses of Polypedates cruciger (common hourglass tree frog)
Source: Front Zool. 2020 Jan 13;17:3. doi: 10.1186/s12983-019-0348-3 (PMC6958743; doi:10.1186/s12983-019-0348-3)
Supplement: Supplementary file 2 — Additional file 2: Table S2. Growth rates of tadpole morphometric characters at different developmental stages. [file 12983_2019_348_MOESM2_ESM.docx]

**Table S2.** Growth rates of tadpole morphometric characters at different developmental stages

| Morphometric character |  | Growth rate (mm day^-1^) | | | |
| --- | --- | --- | --- | --- | --- |
|  | Treatment | | Gosner stage 36-39 | Gosner stage 42-46 | Initial rate |
| Total body length, TBL | Control | | 0.358 | 0.166 | 1.106 |
|  | ETem32 | | 0.123 | -0.105^†^ | 0.839 |
|  | ETem34 | | 0.114 | -0.025^†^ | 1.388 |
|  | ECO2 | | 0.130 | 0.782 | 2.134 |
|  |  | |  |  |  |
| Snout-vent length (SVL) | Control | | 0.154 | 0.122 | 0.279 |
|  | ETem32 | | 0.159 | 0.130 | 0.248 |
|  | ETem34 | | 0.027 | -0.057^†^ | 0.321 |
|  | ECO2 | | 0.409 | 0.209 | 0.729 |
|  |  | |  |  |  |
| Tail length (TL) | Control | | 0.204 | 0.044 | 0.827 |
|  | ETem32 | | 0.200 | 0.086 | 0.558 |
|  | ETem34 | | 0.060 | -0.220^†^ | 0.104 |
|  | ECO2 | | 0.409 | 0.527 | 1.723 |
|  |  | |  |  |  |
| Body width (BW) | Control | | 0.051 | 0.028 | 0.138 |
|  | ETem32 | | 0.010 | -0.030^†^ | 0.135 |
|  | ETem34 | | 0.055 | 0.027 | 0.153 |
|  | ECO2 | | 0.105 | 0.065 | 0.169 |

**Note:** Control – Ambient CO_2_ (water pH=7) and water temperature at 29 ± 1^o^C; ETem32 – Water temperature elevated to 32 ± 0.5^o^C; ETem34 – Water temperature elevated to 34 ± 0.5^o^C. ECO2 – CO_2_ bubbled to water to maintain pH at 5.5-5.6. ^†^As the polynomial functions were in the declining phase in the fitted polynomial functions (Fig. 2), morphometric growth rates in these treatments were effectively zero.
