# Supplementary material for: The impact of elevated temperature and CO2 on growth, physiological and immune responses of Polypedates cruciger (common hourglass tree frog)
Source: Front Zool. 2020 Jan 13;17:3. doi: 10.1186/s12983-019-0348-3 (PMC6958743; doi:10.1186/s12983-019-0348-3)
Supplement: Supplementary file 3 — Additional file 3: Table S3. Significance of contrasts comparing cumulative tadpole mortality in elevated CO2 with that of other treatments. [file 12983_2019_348_MOESM3_ESM.docx]

**Table S3.** Significance of contrasts comparing cumulative tadpole mortality in elevated CO_2_ with that of other treatments

| Contrast | Weeks after hatching | | | |
| --- | --- | --- | --- | --- |
|  | 1 | 2 | 3 | 4 |
| ECO2 vs Control | ns | ns | ns | ns |
| ECO2 vs ETem32 | ns | ns | ns | ns |
| ECO2 vs ETem34 | ns | 0.0138 | 0.0285 | 0.0444 |
| ECO2 vs (ETem32, ETem34) | ns | <0.0001 | ns | ns |

**Note:** Significance of these contrasts was tested in analyses of variance carried out separately for each week using PROC GLM of SAS.
