# Supplementary material for: The impact of elevated temperature and CO2 on growth, physiological and immune responses of Polypedates cruciger (common hourglass tree frog)
Source: Front Zool. 2020 Jan 13;17:3. doi: 10.1186/s12983-019-0348-3 (PMC6958743; doi:10.1186/s12983-019-0348-3)
Supplement: Supplementary file 4 — Additional file 4: Table S4. Significance of contrasts comparing cumulative tadpole mortality in elevated temperatures with that of others. [file 12983_2019_348_MOESM4_ESM.docx]

**Table S4.** Significance of contrasts comparing cumulative tadpole mortality in elevated temperatures with that of others

| Contrast | Weeks after hatching | | | | | | | | | | |
| --- | --- | --- | --- | --- | --- | --- | --- | --- | --- | --- | --- |
|  | 1 | 2 | 3 | 4 | 5 | 6 | 7 | 8 | 9 | 10 | 11 |
| Control vs ETem32 | ns | ns | ns | ns | ns | ns | ns | ns | ns | ns | ns |
| Control vs ETem34 | ns | 0.0098 | 0.0116 | 0.0217 | 0.0154 | 0.0088 | <0.0001 | 0.0002 | 0.0001 | <0.0001 | <0.0001 |
| ETem32 vs ETem34 | ns | 0.0285 | 0.0285 | 0.0444 | 0.0238 | 0.0132 | 0.0001 | 0.0003 | 0.0001 | <0.0001 | <0.0001 |
| Control vs (ETem32, ETem34) | ns | 0.0381 | 0.0475 | 0.0815 | 0.0576 | 0.0376 | 0.0005 | 0.0013 | 0.0007 | 0.0002 | <0.0001 |

**Note:** Significance of these contrasts was tested in analyses of variance carried out separately for each week using PROC GLM of SAS.
