# Supplementary material for: The impact of elevated temperature and CO2 on growth, physiological and immune responses of Polypedates cruciger (common hourglass tree frog)
Source: Front Zool. 2020 Jan 13;17:3. doi: 10.1186/s12983-019-0348-3 (PMC6958743; doi:10.1186/s12983-019-0348-3)
Supplement: Supplementary file 7 — Additional file 7: Plate S1. Lytics zones on gel plates 48 h after innoculation. Con - Ambient CO2 (water pH = 7) and water temperature at 29 ± 1 °C; ECO2 – CO2 bubbled to water to maintain pH at 5.5–5.6. R1 – R4 – Replicates. [file 12983_2019_348_MOESM7_ESM.pdf]

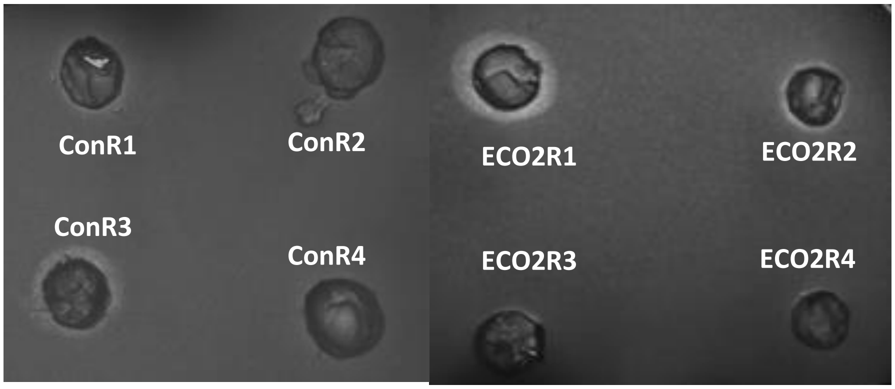

Plate S1. Lytics zones on gel plates 48 hours after innoculation. Con - Ambient CO_2 (water pH=7) and water temperature at 29 ± 1°C; ECO2 – CO_2 bubbled to water to maintain pH at 5.5-5.6. R1 – R4 – Replicates.
